# Mapping and modeling the genomic basis of differential RNA isoform expression at single-cell resolution with LR-Split-seq

**DOI:** 10.1186/s13059-021-02505-w

**Published:** 2021-10-07

**Authors:** Elisabeth Rebboah, Fairlie Reese, Katherine Williams, Gabriela Balderrama-Gutierrez, Cassandra McGill, Diane Trout, Isaryhia Rodriguez, Heidi Liang, Barbara J. Wold, Ali Mortazavi

**Affiliations:** 1 Department of Developmental and Cell Biology, University of California, Irvine, Irvine, CA 92697 USA; 2 Center for Complex Biological Systems, University of California, Irvine, Irvine, CA 92697 USA; 3 Division of Biology, California Institute of Technology, Pasadena, CA 91125 USA

## Abstract

The rise in throughput and quality of long-read sequencing should allow unambiguous identification of full-length transcript isoforms. However, its application to single-cell RNA-seq has been limited by throughput and expense. Here we develop and characterize long-read Split-seq (LR-Split-seq), which uses combinatorial barcoding to sequence single cells with long reads. Applied to the C2C12 myogenic system, LR-split-seq associates isoforms to cell types with relative economy and design flexibility. We find widespread evidence of changing isoform expression during differentiation including alternative transcription start sites (TSS) and/or alternative internal exon usage. LR-Split-seq provides an affordable method for identifying cluster-specific isoforms in single cells.

**Supplementary Information:**

The online version contains supplementary material available at 10.1186/s13059-021-02505-w.

## Introduction

Alternative transcript isoform expression is a major regulatory process in eukaryotes that includes differential TSS (transcription start site) selection, RNA splicing, and TES (transcription end site) selection. These differential choices sculpt the transcriptome and its resulting proteome during development, across cell types and in disease states. However, it has proved challenging to fully capture and quantify isoform regulation by standard short-read RNA-seq because of the ambiguity it leaves in mapping the transcript termini and full-length exon connectivity that define each mature isoform.

In recent years, long-read RNA sequencing technologies have emerged as a powerful alternative for transcript-level identification and quantification by going beyond the level of exon usage to simultaneously identify novel isoforms with alternative TSSs, TESs, and exon combinations. Furthermore, long-read RNA-seq has been adapted to single-cell sequencing using high-throughput microfluidics-based methods [1–4]. Some of these studies sequenced the same cells with both PacBio and Illumina technologies and relied on short-read gene quantification to cluster and characterize cell types, while using the long reads to identify full-length isoforms [2, 4]. However, these prior approaches used expensive equipment, such as microfluidics platforms, and/or applied very high amounts of long-read sequencing whose expense limits routine and extensive application.

Differential RNA isoforms discriminate cell types within complex tissues and, within cell types such as neurons, can further distinguish functionally distinct cell subpopulations [5, 6]. Isoform choice can even distinguish individual neurons of the same “type” from each other [7, 8]. Transcript isoforms also discriminate developmental stages and disease states [9]. In vertebrate systems, differential isoform regulation through development has long been appreciated, and in some disease states such as type 1 myotonic dystrophy, fetal or neonatal stage isoforms of *Tnnt2, Atp2a1* (*Serca1*), and *Ldb3* (*Zasp*) are inappropriately expressed [10–12]. In addition, several studies have characterized the diversity of gene expression within the population of nuclei from myotubes [13, 14]. This prior work on skeletal muscle provides known instances of isoform choices that we can use to benchmark new methods for transcriptome profiling, while at the same time posing unanswered questions that require single-cell or single-nucleus long-read data such as nuclear specialization within myotubes.

In vitro differentiation of the myogenic C2C12 cell line from proliferating, mononucleated myoblasts to multinucleated myotubes is a widely used model of myogenesis due to transcriptional and morphological similarities to the in vivo process [15]. A subset of cells under differentiation promoting conditions remain mononucleated and are called MNCs [16, 17]. In adult muscle tissue, satellite cells are mononucleated muscle stem cells that can be stimulated to proliferate and differentiate to drive muscle repair [18]. Expression of the satellite cell marker gene *Pax7* decreases as satellite cells are activated into proliferating myoblasts, while expression of myogenic regulatory factors (MRFs) such as *Myod1* and *Myog* increase and promote myogenesis [18]. Satellite cells undergo asymmetric divisions to produce future *Pax7* negative, MRF-positive myoblasts and to self-renew *Pax7*-positive, MRF-negative satellites [19]. In addition to major transcriptional changes during myogenesis, C2C12 differentiation exhibits substantial changes, both qualitative and quantitative, in splice isoforms [20]. For example, *Pkm* undergoes an isoform switch during C2C12 differentiation that results in two distinct isozymes of the gene, PMK2 and PKM1, which include mutually exclusive exons 9 and 10 respectively [21]. Proliferating C2C12s express both isoforms of beta-tropomyosin (*Tpm2*), including exon 6a or exon 6b, but expression of the 6b isoform increases substantially during differentiation [21].

Here, we combine combinatorial barcoding of individual C2C12 cells and nuclei using the Split-seq strategy [22] with long-read sequencing (LR-Split-seq) to investigate isoform changes during differentiation. We first examined the technical differences between LR-Split-seq random hexamer and oligo-dT priming strategies as well between single cell and single nucleus. We compared the performance of LR-Split-seq to bulk long-read RNA-seq, and further compared the clusters recovered from LR-Split-seq to those from short-read sequencing for the same cells, as well as a companion dataset of 37,000 cells to show that long-read single-cell transcriptomes produce similar results to short-read that can be readily integrated. We then leveraged LR-Split-seq results to identify and quantify TSSs in order to perform differential TSS testing and examine TSS usage between single-cell clusters. Finally, we integrated the resulting TSS expression from LR-Split-seq with matching single-cell ATAC-seq to quantify the extent of coordinated single-cell chromatin accessibility.

## Results

### Comparing oligo-dT versus random hexamer primed long-read data

**Fig. 1:**
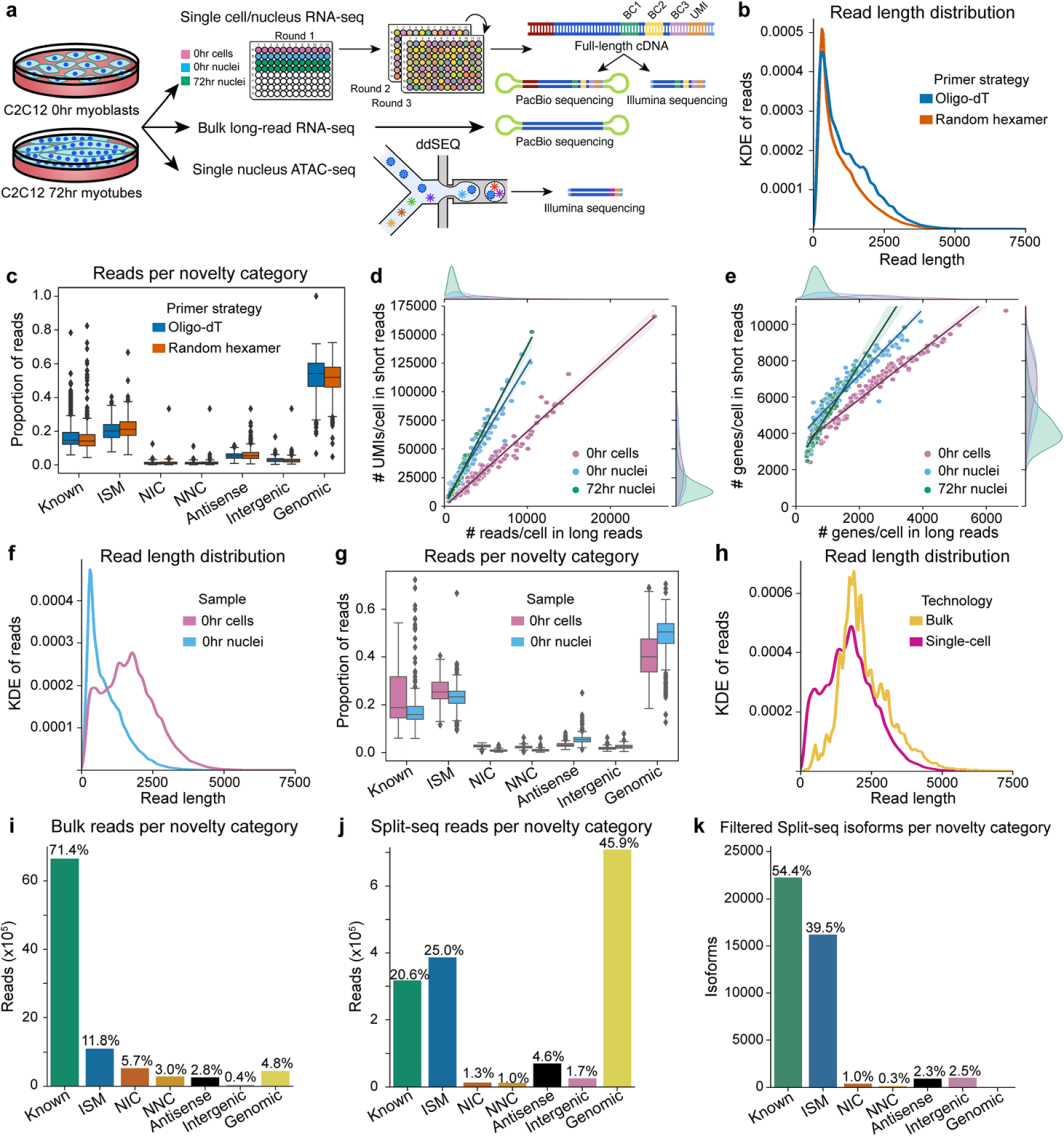
Technical comparisons in LR-Split-seq and bulk long-read RNA-seq. **a** Schematic diagram of experimental design. Single cell/nucleus LR-Split-seq, short-read Split-seq, bulk long-read RNA-seq, and single nucleus ATAC-seq were performed on C2C12 0 h myoblasts and 72 h differentiating cells. The same single-cell/UMI-barcoded cDNA was used in both short-read and long-read sequencing. **b** Kernel density estimation (KDE) of read length distribution of oligo-dT primed reads (blue) compared to random hexamer primed reads (orange). **c** Proportion of oligo-dT/random hexamer reads in each cell for each novelty category. **d** Comparison of number of reads and **e** genes detected between short and long reads. Cells are labeled by sample type (0 h cells in pink [regression m = 1.4 and 6.5 in genes and reads respectively], 0 h nuclei in blue [regression m = 1.8 and 12.0 in genes and reads respectively], and 72 h nuclei in green [regression m = 2.7 and 13.9 respectively]) and marginals on the top and right indicate their distributions. **f** KDE read length distribution of 0 h cells (pink) compared to 0 h nuclei (blue) reads, not including genomic reads. **g** Proportion of 0 h cell (pink)/nuclei (blue) reads per cell/nucleus per novelty category. **h** KDE read length distribution of bulk long reads (yellow) compared to single-cell long reads (magenta), not including genomic reads. **i** Unfiltered reads per novelty category in bulk long-read data and **j** LR-Split-seq data. **k** Filtered isoforms per novelty category across all cells in LR-Split-seq data

Split-seq uses a combination of oligo-dT and random hexamer primers in order to decrease the 3′ bias that dominates other single-cell RNA-seq methods that prime only with oligo-dT [22]. These methods are designed to perform 3′ end counting for sequenced genes but they give little or no information about the rest of the transcript. In contrast, when Split-seq is conventionally performed with short reads, the random priming feature should, in the ideal instance, provide comprehensive information about the entire body of the transcript. However, this benefit in the short-read format is expected to have a different and unfavorable effect in long-read data. The extent and character of effects from internal priming will depend on multiple protocol variables (e.g., relative amounts of oligo-dT versus random hexamers, substrate RNA integrity) and on filtering steps in the subsequent informatic pipeline. We therefore began by testing the impact of priming strategy on the LR-Split-seq data. We collected proliferating C2C12 myoblasts (0 h) as both whole cells and nuclei, then differentiated the remainder into myotubes over 3 days to recover 72-h differentiated nuclei (Methods). We labeled a total of approximately 37,000 cells/nuclei from the three samples using the Split-seq combinatorial barcoding strategy. We then built a sublibrary of 1000 cells for sequencing by PacBio as well as Illumina (Fig. 1A). The LR-Split-seq data was first debarcoded and demultiplexed using our LR-splitpipe pipeline (Additional file 1: Fig. S1A) (Methods).

We then analyzed the reads with TALON [23], which is designed to assign long reads to their transcripts of origin and to identify new transcripts (Additional file 1: Fig. S1B-C, Additional file 2: Table S1) (Methods). TALON’s long-read RNA-seq annotation then assigns each read to a category that specifies whether the read matches a known transcript in the reference transcriptome GTF file, or if it represents a novel transcript [23, 24]. Novel reads and transcripts are further broken down by how they are novel compared to the reference annotation. Incomplete splice match (ISM) transcripts contain a subsection of an annotated transcript but do not extend all the way to the annotated 3′ or 5′ end. Novel in catalog transcripts (NIC) contain a new combination of exons that are all present in the reference annotation. Novel not in catalog transcripts (NNC) contain at least one splice site that is not present in the reference annotation. Antisense transcripts come from the opposite strand of a gene, and intergenic transcripts are from regions of the genome with no annotated genes. Finally, genomic transcripts overlap genes but do not share any known splice junctions with those in the annotation. Genomic transcripts are often monoexonic, short, or contain intronic regions.

Random hexamer priming is expected to start within the body of a transcript rather than the 3′ polyA tail where oligo-dT primers hybridize, though intronic A-rich runs are known to serve as additional start points for oligo-dT priming [25]. This mixed priming strategy, as it is currently implemented in the Split-seq commercial platform, produced remarkably little difference in the final LR-Split-seq read length distribution from the two primer types (Fig. 1B, C). The distribution of reads per TALON category showed a slightly higher proportion of incomplete splice match (ISM) reads per cell from the random hexamer priming strategy versus the oligo-dT priming strategy (Fig. 1C). We speculate that the high fraction of oligo-dT primed reads per cell that begin at internal sites (~ 60%) accounts for the overall similarity of random hexamer primed reads in length profiles and genes detected.

### Single nuclei compared with single cells for LR-split-seq

We compared single-cell versus single-nucleus LR-split-seq. Overall, more reads and genes were recovered from whole cells versus nuclei for both long- and short-read data, which is expected because cytoplasmic transcripts are left behind during nuclear extraction, making the nuclei less sensitive than whole cells (Fig. 1D, E). When comparing only 0 h cells with companion nuclei, we observe shorter read lengths in the nuclei (Fig. 1F). And as expected, we also see a larger proportion of genomic reads per cell/nucleus in nuclei compared to cells (Fig. 1G). These nuclear genomic reads could result from the enrichment of intronic RNA in the nucleus which would explain the lack of splice junctions.

Comparing LR-Split-seq of whole cells with bulk long-read RNA-seq for myoblasts, we found that the LR-Split-Seq is modestly shorter than bulk long-read data (Fig. 1H, Additional file 2: Table S2). Bulk reads have an average mean length of 2274 bp and a peak from the kernel density estimate (KDE) distribution of 1875 bp, versus an average mean length of 1735 bp and a KDE peak of 1791 bp for LR-Split-seq non-genomic reads from whole cells (Fig. 1H, Additional file 2: Table S2). The LR-Split-seq reads also had more genomic and incomplete splice match (ISM) reads than the bulk data (Fig. 1I, J). These differences are in line with expectations, given other differences in details of the bulk protocol (Methods). Nevertheless, after strictly filtering our novel transcripts with TALON, we retain 40,982 of the original 466,078 originally identified isoforms which represent 34.8% of reads and 34.5% of UMIs. The majority of transcript models are known transcripts annotated in GENCODE (Methods, Additional file 1: Fig. S1D, Fig. 1K). The observed read length differences between LR-Split-seq and bulk is reflected in the genes and transcripts that are uniquely detected in the bulk or LR-Split-seq. Transcripts detected only in bulk transcriptomes were likely to be longer, whereas transcripts detected only in LR-Split-seq data were enriched for shorter length (Additional file 1: Fig. S1E, S1F). Due to overall longer read length in bulk long reads, these data were more likely to have multiple exons than LR-Split-seq (Additional file 1: Fig. S1G). We conclude that the read length profile of known reads in single-cell LR-Split-seq is quite similar to bulk long reads, given protocol differences. This suggests to us that the overall shorter lengths in single-nucleus versus whole-cell LR-split-seq are of biological origin, likely driven by underlying differences between cytosolic RNA, which is rich in mature mRNA versus nuclear RNA, which contains mature mRNA but in lower proportions.

### LR-Split-seq and bulk long-read RNA-seq detect similar gene sets

**Fig. 2:**
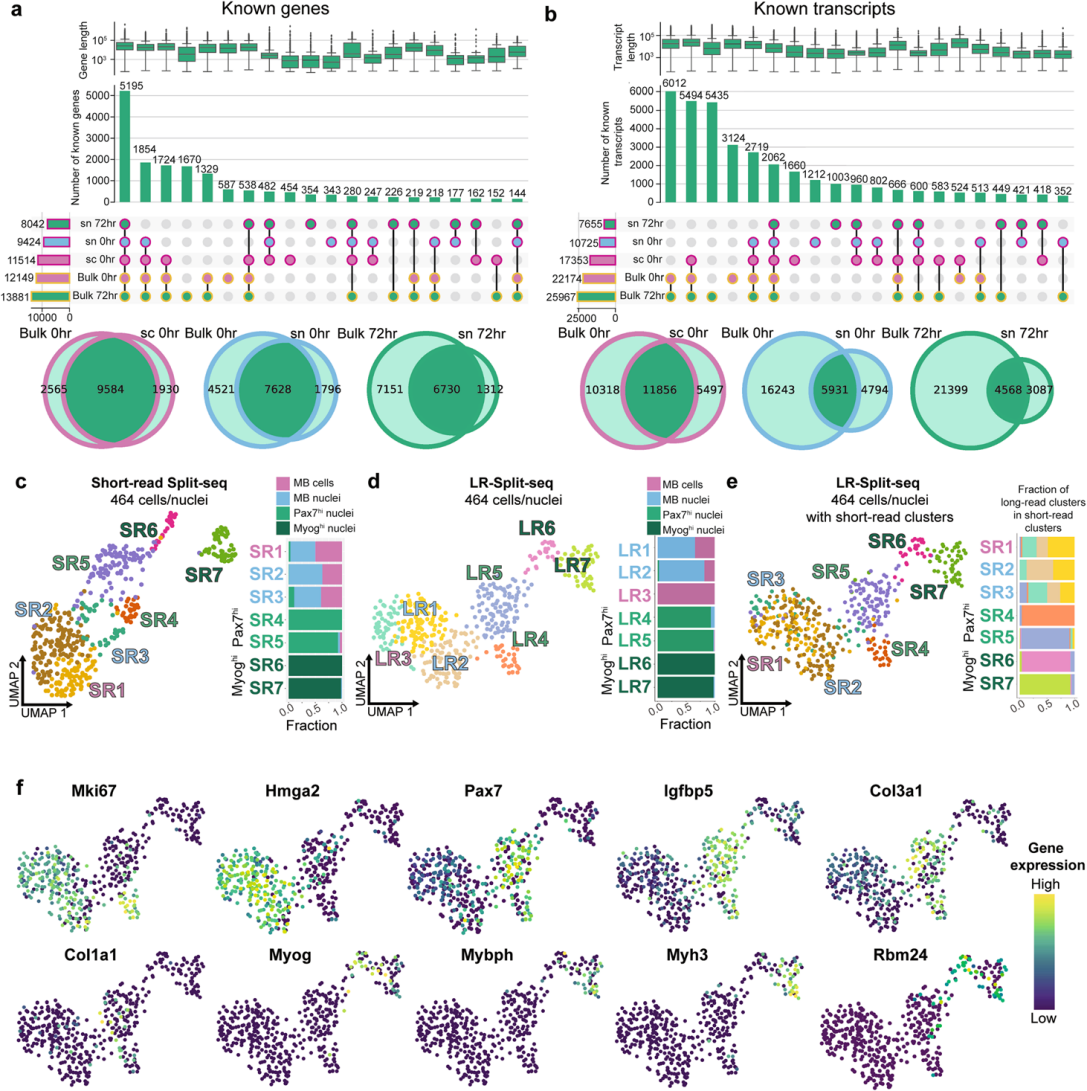
LR-Split-seq in C2C12 0 h and 72 h samples recapitulates results from companion bulk and standard short-read Split-seq. **a** Upset plots of known genes found in bulk compared to LR-Split-seq data across all samples. Bars on the left indicate set size, circles indicate combinations of samples, and bars on top indicate the number of genes found in each combination (first 20 combinations shown). Outline colors indicate technology (bulk in yellow, single-cell or single-nucleus in magenta) and fill colors indicate sample type (72 h nuclei in green, 0 h nuclei in blue, and 0 h cells in pink for single-cell data; 72 h in green, 0 h in pink for bulk data). Box plots above indicate gene length distribution for each intersection. Venn diagrams below summarize the overlaps between bulk (left) and single-cell or single-nucleus (right), for each sample type. Sample type is indicated by outline color. **b** Upset plot and Venn diagrams of known transcripts found in bulk data and LR-Split-seq data (first 20 combinations shown). **c** UMAP of 464 short-read Split-seq cells/nuclei labeled by 7 Leiden clusters (S) and breakdown of cell type per cluster: 110 0 h cells (pink), 145 0 h nuclei (blue), and 209 72 h nuclei (*Pax7*^*hi*^ in green and *Myog*^*hi*^ in dark green). **d** UMAP of 464 LR-Split-seq cells/nuclei using gene-level data labeled by 7 Leiden clusters (LR) and **e** Leiden cluster ID of matching short-read data (SR) shown in **c**, as well as long-read cluster makeup of each short-read cluster. **f** Expression of marker genes, dark blue = lowly expressed, yellow = highly expressed

Despite differences in transcript length and novelty classification between bulk long-read RNA-seq and LR-Split-seq, we detected 9584 known genes in both bulk and single-cell LR-Split-seq, with 5195 of these shared across all assays and sample combinations (Fig. 2A). These results demonstrate the gene detection sensitivity of LR-Split-seq. The next largest intersections contain > 1500 genes recovered in all but the single-nucleus data which is likely due to the relative loss of cytoplasmic transcripts from the nuclear preparation. Genes detected in LR-Split-seq but not in the companion bulk RNA-seq tend to be short and are enriched for short RNA biotypes such as snoRNAs and miRNAs, while genes detected solely in bulk data are enriched for protein coding genes (Additional file 2: Table S2). A plausible explanation is that Split-seq’s random hexamer priming captured these transcript types whereas the bulk method, which uses oligo-dT priming exclusively, preferentially captured polyadenylated transcripts. We also examined the overlap between filtered novel transcript models from the known, NIC, and NNC novelty categories in bulk and LR-Split-seq (Fig. 2B). While the vast majority of novel transcript models were only reproducible between bulk replicates, 251 NIC transcripts and 61 NNC transcripts were reproducible in at least one bulk and one LR-Split-seq sample (Additional file 1: Fig. S1H, S1I). These represent isoforms that are most likely to be real, though not previously annotated. Assuming that only the novel NIC and NNC transcripts found in bulk are real gives us a true positive rate (TPR) of 0.79 for NIC and 0.59 for NNC. We note that the calculated TPR for NIC based on the bulk is higher than for known transcripts detected by LR-Split-seq (0.71) and that it is certainly possible that an additional subset of the NIC/NNC isoforms discovered in LR-split-seq were missed in the bulk because they are lowly expressed.

### LR-Split-seq recapitulates cell classifications recovered from short-read Split-seq

Overall, we recovered 110 0 h myoblast cells, 145 0 h myoblast nuclei, and 209 72 h differentiating nuclei (464 cells total) that passed short-read QC thresholds as well as an additional requirement of ≥ 500-long reads per cell in the 1000-cell library (Methods) (Additional file 1: Fig. S2A-E). Leiden clustering based on short-read sequencing of the 464 cells/nuclei yielded 7 clusters (SR1-SR7). We observed mixed populations of 0 h myoblast cells and nuclei in clusters SR1-SR3, while the 72 h differentiating nuclei clustered in SR4-SR7. This overall structure is consistent with differentiation playing a dominant role in the UMAP structure, while differences between nuclei versus whole cells from the 0 h samples were minor by comparison (Fig. 2C). Additional patterns in the dataset that agree with known biology in the system include expression of the satellite cell marker gene Pax7, which is expressed mainly in 72 h clusters SR4 and SR5, while the key myogenic transcription factor *Myog* (myogenin) is expressed mainly in 72 h clusters SR6 and SR7 (Additional file 1: Fig. S3A). An independent Leiden clustering performed using the LR-Split-seq data for the same 464 cells proved very similar to the companion short-read clustering with 7 clusters (LR1-LR7) in which the myoblast progenitor cells/nuclei are in clusters LR1-LR3 while the differentiating sample gives rise to clusters LR4-LR7 (Fig. 2D). This UMAP again separates the latter group into *Pax7*^*hi*^ (LR4, LR5) and contrasting *Myog*^*hi*^ sets (LR6, LR7), with the latter expressing additional downstream markers of myocyte differentiation. Color-coding cells in the long-read UMAP according to the cluster identity from the companion short-read data showed high concordance of clusters LR4-LR7 with SR4-SR7 (Fig. 2E). The myoblast progenitor clusters SR1-SR3 and LR1-LR3 also agree, although the short-read clusters were more mixed between 0 h cells and nuclei.

We furthermore compared our short- and long-read cells using independent RNA velocity analyses using Velocyto [25]. We found when comparing the ratio of spliced to unspliced reads in both read formats that there was typically a higher proportion of spliced reads detected in the short reads per cell versus the long reads, which may be due to the overall higher probability of sequencing an intronic region per read in long reads (Additional file 1: Fig. S3B). However, the difference is minor and the resulting independent trajectories are very similar between the short and long read experiments (Additional file 1: Fig. S3C). We investigated gene expression patterns for additional known marker genes across the cells and nuclei between the short-read and long-read clusters (Fig. 2F). Most notably, *Mybph*, *Myh3*, and *Mef2c* are highly expressed in a subset of 72 h nuclei that make up cluster LR7, whereas *Myog* is expressed in both clusters LR6 and LR7 of 72 h nuclei (Fig. 2F, Additional file 1: Fig. S3A). Similar to the short-read data, *Pax7* is present in both 0 h and 72 h clusters, but it is most highly expressed in clusters LR4 and LR5 (Fig. 2F). We also capture similar expression patterns in short-read and long-read *Pax7*^*hi*^ 72 h subclusters as indicated by *Igfbp5*, *Col3a1*, and *Col1a1* (Fig. 2F, Additional file 1: Fig. S3A). Due to the consistent expression patterns of known marker genes across both technologies, we postulate that *Myog*^*hi*^ clusters SR6, SR7, LR6, and LR7 are mainly nuclei originating from fused, multinucleated myotubes or mononucleated myocytes on their way toward fusion, while the *Pax7*^*hi*^ clusters SR4, SR5, LR4, and LR5 are nuclei distinct from both myoblasts and the 72 h *Myog*^*hi*^ nuclei.

We examined the isoform complexity of each cell by counting the number of genes that express multiple isoforms from the same single cell or nucleus. Only one isoform was typically detected per gene in each cell. The number of genes expressing more than one isoform is a linear function of read depth per cell, suggesting that deeper sequencing will increase isoform complexity per gene (Additional file 1: Fig. S3D). Furthermore, we noticed a clear difference in the relationship between isoform complexity and read depth when comparing the single cells with single nuclei where the nuclei of increasing read depth do not display a similar large increase in isoform complexity as do the cells (Additional file 1: Fig. S3D). One explanation for this is that LR-Split-seq of a nucleus captures a snapshot of its immediate state of splicing, whereas LR-Split-seq in cells captures the sum of different isoforms produced and exported to the cytoplasm over a longer period of time. If correct, the implication is that splicing within the nucleus is transiently biased for one pattern, and conceivably for one allele, the identity of which changes dynamically.

We additionally performed isoform-switching tests across three identified groups of clusters: 0 h myoblast (MB) cells (LR1-LR3), 72 h Pax7hi nuclei (LR4-LR5), and 72 h Myoghi nuclei (LR6-LR7), with a corrected *p* value cutoff from a chi-squared test of 0.05 and a change in percent isoform usage cutoff of ≥ 10% [4] (Methods). We recovered statistically significant isoform-switching genes that have been previously observed in differentiating C2C12s, such as*Tpm2* (*Adj. P* = 1.06 × 10^−5^ MB vs. 72 h *Myog*^*hi*^) and *Pkm* (*Adj. P* = 2.57 × 10^−11^ MB vs. 72 h *Pax7*^*hi*^; *Adj. P* = 2.98 × 10^−7^ MB vs. 72 h *Myog*^*hi*^). The *Tpm2* locus specifically shows an increase in expression of and preference for isoforms containing exon 6b in the differentiated nuclei as previously characterized in C2C12s as visualized with Swan (Additional file 1: Fig. S4A) [21, 26]. We detect distinct *Pkm* isoforms with mutually exclusive exons 9 and 10 that correspond to the isozymes PKM1 and PKM2. The myoblasts tend to produce the exon 10-containing isoform (Pkm-201) over the major exon 9-containing isoform (Pkm-202), whereas the differentiated nuclei seem to equally produce Pkm-201 and Pkm-205, which has an alternative TES (Additional file 1: Fig. S4B). We found 21 significant isoform-switching genes between MB nuclei and 72 h *Pax7*^*hi*^ nuclei as well as 14 significant isoform-switching genes between MB nuclei and 72 h *Myog*^*hi*^ nuclei (Additional file 2: Table S3, Additional file 2: Table S4).

### C2C12s have distinct *Pax7*^*hi*^ subpopulations following differentiation

**Fig. 3:**
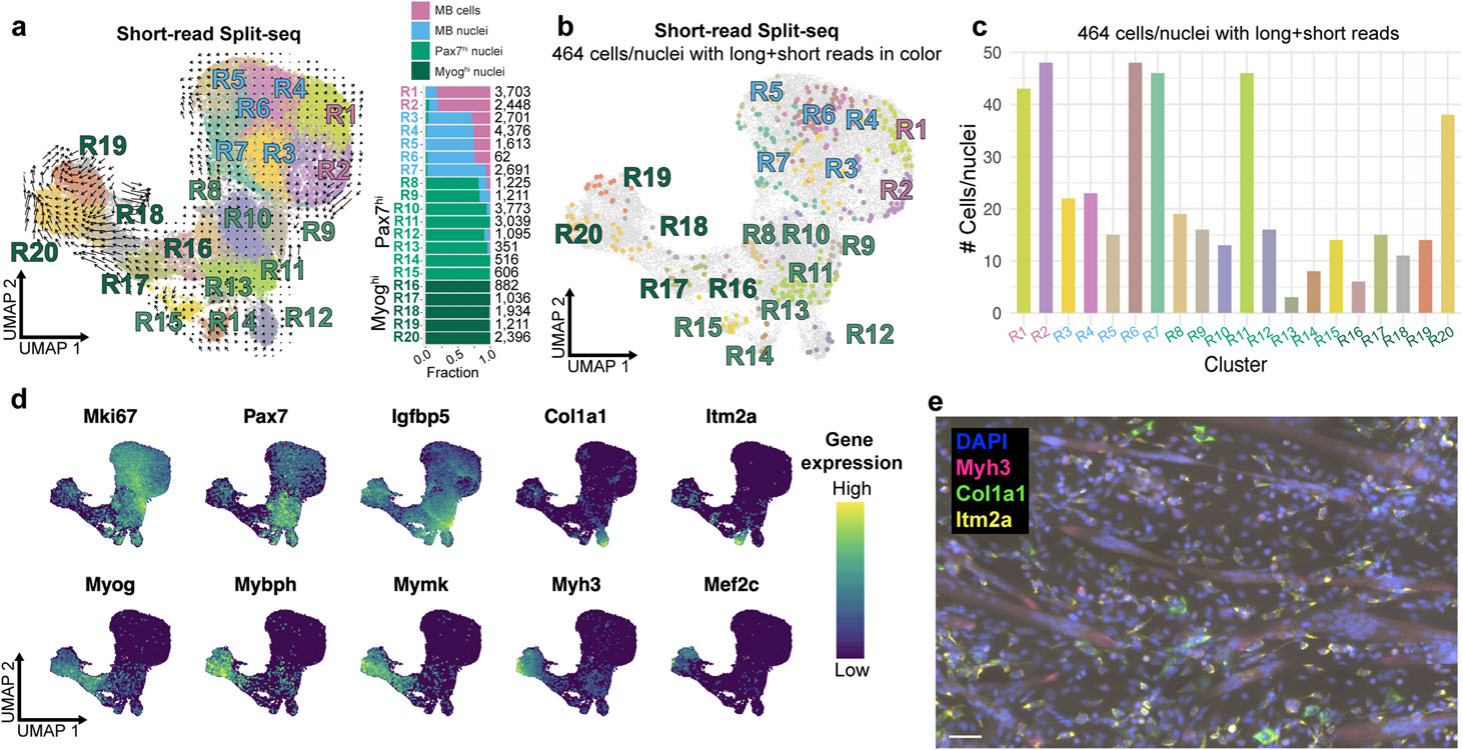
Short-read Split-seq analysis. **a** UMAP of 36,869 short-read Split-seq cells/nuclei labeled by 20 Leiden clusters (R) with RNA velocity field trajectories and breakdown of cell type per cluster with number of cells per cluster: 7797 0 h myoblast cells (pink), 10,194 0 h myoblast nuclei (blue), 18,878 72 h nuclei (*Pax7*^*hi*^ in green and *Myog*^*hi*^ in dark green). **b** UMAP of short-read Split-seq cells/nuclei with the 464 cells with matching long reads in color corresponding to R1-R20. **c** Histogram of the number of the 464 cells/nuclei per R1-R20. **d** Distribution of expression of marker genes; dark blue = lowly expressed, yellow = highly expressed. **e** Visualization of transcripts in mononucleated cells and myotubes at the 72 h differentiation timepoint. Blue = DAPI, pink = *Myh3*, green = *Col1a1*, yellow = *Itm2a*. Scale bar: 50 μm

We confirmed the presence of distinct *Pax7*^*hi*^ and *Myog*^*hi*^ clusters by short-read sequencing of an extended set of cells and nuclei from the same labeled pool, comprised of six additional 9000-cell sublibraries on top of the 1000-cell sublibrary with matching long reads (Methods). After filtering, we recovered 36,869 total cells/nuclei from all seven sublibraries, including the 464 cells/nuclei with both short and long reads (Additional file 1: Fig. S2A-E). The 7797 myoblast cells, 10,194 myoblast nuclei, and the 18,878 differentiating condition nuclei clustered primarily by differentiation state (Fig. 3A). The progenitor states form one main group in UMAP space that slightly separates cells and nuclei, while the differentiating nuclei extend outward in a spectrum with several smaller groups (Fig. 3A). Of the 20 clusters identified by Leiden clustering, 7 consist mostly of myoblast cells/nuclei while 13 are mainly differentiating nuclei (Fig. 3A) (Methods). Out of the 13 72 h clusters, 8 are *Pax7*^*h*i^ and the other 5 are *Myog*^*hi*^, which is consistent with results from the 464 cells alone (Fig. 3A). Accordingly, cells from each of the 20 clusters are represented by both short and long reads in the 464-cell subset (Fig. 3B, C). We assign these clusters to the cells we recovered with long reads to better inform the cellular identities with high resolution (Additional file 1: Fig. S5A). For example, a small subset of 12 cells out of 105 total cells in cluster SR5 belong to cluster R12, which is distinguished by high expression of *Col1a1* (Fig. 3D). Genes critical for cell cycle phases G1 and S such as *Cdk2* and *Pcna* are highly expressed in MB cluster R1, while G2 and M phase marker gene *Top2a* is highly expressed in MB clusters R2 (made up of mostly cells) and R3 (made up of mostly nuclei) as well as *Pax7*^*hi*^ cluster R9 (Additional file 1: Fig. S5B) [27–29]. *Myog* is expressed throughout multinucleated myotubes as well as in some mononucleated cells that are likely to be pre-fusion myocytes (Additional file 1: Fig. S5C). *Myog* and myogenic marker gene *Mybph* are highly expressed in clusters R16, R17, R18, and R20, indicating that these nuclei most likely belong to committed myocytes and myotubes (Fig. 3D). RNA velocity analysis, which uses the ratio of intronic (unspliced) and exonic (spliced) reads to predict the transcriptional trajectory of cells, reveals a lineage from clusters R17 and R18 toward clusters R19 and R20. R19 and R20 express terminal myogenic marker genes such as *Myh3*, *Mef2c*, *Tnnt2*, and *Neb* (Fig. 3D, Additional file 1: Fig. S5D) (Methods). Of the 8 co-adjacent *Pax7*^*hi*^ clusters (R8, R9, R10, R11, R12, R13, R14, and R15), some also express cluster-discriminating genes such as *Igfbp5* (cluster R11), *Col1a1* (cluster R12), and *Itm2a* (cluster R14) (Fig. 3D, Additional file 1: Fig. S5D). We validated differential cluster-specificity of marker genes using spatial transcriptomic profiling of *Col1a1* (cluster R12), *Itm2a* (cluster R14) and *Myh3* (cluster R20), which showed patterns fully consistent with the Split-seq data (Fig. 3E). Imaging also confirmed that *Pax7*^*hi*^ subcluster marker genes are expressed in MNCs rather than in the multinucleated myotubes that they surround (Fig. 3E). *Myh3* is expressed throughout multinucleated myotubes but less so in mononucleated cells. *Pax7*^*hi*^ MNCs appear to either express *Col1a1* or *Itm2a,* consistent with their mutually exclusive marking of clusters R12 and R14 (Fig. 3D, E).

We observed heterogeneous populations of differentiating cells representing cell populations and states that are involved in adult muscle tissue repair. Clusters R10 and R11 express *Igfbp5*, which promotes muscle differentiation, and *Nfix*, which controls timing of regeneration by repressing myostatin (Fig. 3D, Additional file 1: Fig. S5D, Additional file 2: Table S5) [30, 31]. Cluster R12, marked by *Col1a1*, *Fn1* (fibronectin), and a number of other collagen genes, may represent a population of previously defined MNCs that can transiently remodel their ECM, which is a process shown to regulate satellite cell numbers in vivo (Fig. 3D, Additional file 1: Fig. S5D, Additional file 2: Table S5) [32, 33]. Cluster R13 expresses *Lix1*, a *Pax7* target gene needed for activated satellite cell proliferation (Additional file 1: Fig. S5D, Additional file 2: Table S5) [34]. Cluster R14, which expresses *Itm2a* and *Pax7*, may be analogous to activated satellite cells (Fig. 3D, Additional file 1: Fig. S5D, Additional file 2: Table S5) [35]. Appropriately, the cluster R14 RNA trajectory tends toward cluster R15 which expresses *Tead1* (*Tef-1*) and *Myog*, which are known to promote muscle differentiation (Additional file 1: Fig. S5E, Additional file 2: Table S5) [36].

### Chromatin accessibility of myogenic marker genes distinguishes *Myog*^*hi*^ and *Pax7*^*hi*^ 72 h nuclei

**Fig. 4:**
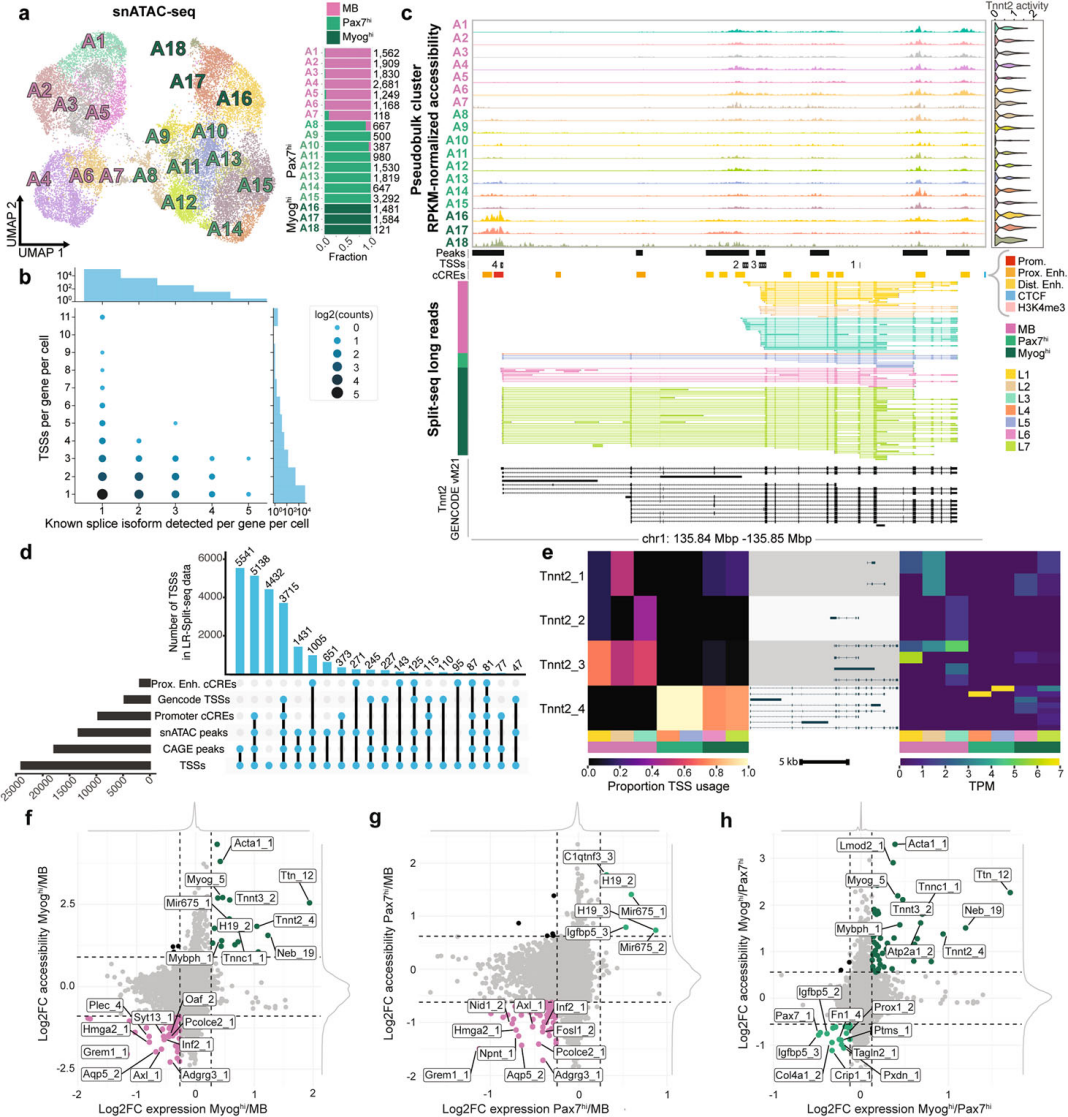
Identification of TSSs from LR-Split-seq and integration with snATAC-seq. **a** UMAP of 23,525 snATAC-seq nuclei labeled by 18 Leiden clusters (A) and breakdown of cell type per cluster with number of cells per cluster on right: 10,508 0 h myoblast nuclei (pink) and 13,017 72 h nuclei (*Pax7*^*hi*^ in green and *Myog*^*hi*^ in dark green). **b** Bubble plot of the number of distinct known splice isoforms per gene per cell compared to the number of distinct TSSs per gene per cell in LR-Split-seq. **c** Track plot of alternative *Tnnt2* TSS usage between 72 h differentiating cells and 0 h myoblasts. From top to bottom: clustered snATAC-seq pseudobulk peaks, merged psuedobulk peaks, TSS regions called from LR-Split-seq, ENCODE cCREs, clustered LR-Split-seq reads used to call TSSs, and comprehensive set of GENCODE vM21. **d** Validation of TSSs found in LR-Split-seq using four external datasets and snATAC-seq pseudobulk peaks (first 20 intersections shown). **e** Left, proportion of TSS-assigned reads in LR-Split-seq clusters from each identified *Tnnt2* TSSs. Right, expression of each TALON filtered *Tnnt2* isoform in LR-Split-seq clusters with corresponding transcript models associated with each *Tnnt2* TSS. **f** Comparison of log2 fold change (LFC) in expression and accessibility across identified TSSs: *Myog*^*h*i^ (+LFC) compared to MB (-LFC), **g** *Pax7*^*hi*^ (+LFC) compared to MB (-LFC), and **h** *Myog*^*hi*^ (+LFC) compared to *Pax7*^*hi*^ (−LFC)

To assess chromatin accessibility in the groups of nuclei we identified with LR-Split-seq, we performed snATAC-seq on matching timepoints. We recovered 23,525 single nuclei from our snATAC-seq experiments following filtering and QC (Additional file 1: Fig. S6A-B), resulting in 18 clusters from Leiden clustering: seven 0 h myoblast clusters and eleven 72 h differentiating clusters (Fig. 4A) (Methods). “Gene activity” in this context refers to a measure of chromatin accessibility of the gene body and 2 kb upstream as a rough estimate of transcriptional activity [37]. We saw that chromatin gene activity patterns in our snATAC-seq UMAP for *Myog* is somewhat similar to scRNA-seq expression patterns, where the *Myog* locus was highly accessible in a subset of differentiated clusters (A16, A17, and A18) (Additional file 1: Fig. S6C). To investigate the agreement between expression and chromatin accessibility for the same time points, 0 h and 72 h, we integrated our short-read Split-seq and snATAC single-cell measurements using Signac (Methods) [38]. This integration mapped Split-seq cells on snATAC-seq nuclei, resulting in predicted snATAC-seq cell types. The predicted Split-seq time point (0 h or 72 h) was mostly accurate, with 96% (10,136 out of 10,508) of true snATAC 0 h nuclei predicted to be 0 h from the expression data and 79% (10,381 out of 13,017) of true snATAC 72 h nuclei predicted as 72 h (Additional file 1: Fig. S6D). When we mapped Split-seq cells grouped by MB (R1-R7), *Pax7*^*hi*^ (R8-R15), and *Myog*^*hi*^ (R16-R20) onto snATAC nuclei, we found that 48% (1502 out of 3148) of nuclei with a *Myog* activity score > 0 were predicted to be *Myog*^*hi*^ and that 27% (5135 out of 18,542) of nuclei with a *Pax7* activity score > 0 were predicted to be *Pax7*^*hi*^ (Additional file 1: Fig. S6D). Unlike our Split-seq RNA data, where we detected high expression of *Pax7* in specific clusters, ATAC-based gene activity scores predicted that *Pax7* would be equally active across all clusters (Additional file 1: Fig. S6E). Taken at face value, this suggests that some differentially expressed genes do not exhibit corresponding changes in promoter chromatin state, as reflected by these activity scores. However, there are several distal peaks ATAC peaks located downstream of *Pax7* whose dynamics are coordinated with the RNA. This suggests, as a working model, that they are regulatory elements governing *Pax7* expression. In contrast, *Myog* and *Mybph* illustrate expected coordinated changes in chromatin accessibility and RNA isoform expression during differentiation (clusters A16-A18) at the TSSs of these genes (Additional file 1: Fig. S6F). For uniform terminology between RNA and DNA data, we label 72 h *Myog*^*low*^ snATAC clusters A8-A15 as *Pax7*^*hi*^. While snATAC can clearly capture changes in chromatin remodeling, the ATAC-only gene activity scores (at least as computed by Signac) do not reflect the *Pax7* expression-level changes that we measure in this system.

As expected, investigation of marker peaks for *Myog*^*hi*^ clusters A16-A18, using a gene annotation method with gene ontology analysis, revealed significant terms such as muscle system process (*P* = 1.55 × 10^−115^), muscle structure development (*P* = 5.77 × 10^−118^), and striated muscle contraction (*P* = 3.87 × 10^−96^) (Methods, Additional file 1: Fig. S6G, Additional file 2: Table S6, Additional file 2: Table S7). In comparison, MB clusters A1-A7 had broad significant terms such as regulation of anatomical structure morphogenesis (*P* = 1.69 × 10^−19^), cell-cell adhesion (*P* = 3.45 × 10^−13^), and cell motility (*P* = 3.89 × 10^−14^) (Additional file 2: Table S6, Additional file 2: Table S7). The significant terms for *Pax7*^*hi*^ clusters A8-A15, in contrast to *Myog*^*hi*^ clusters, were extracellular matrix organization (*P* = 1.23 × 10^-9^), extracellular structure organization (*P* = 1.35 × 10^-9^), and blood vessel morphogenesis (*P* = 3.92 × 10^−9^) (Additional file 2: Table S6, Additional file 2: Table S7). Most marker peaks defining the *Myog*^*hi*^ clusters are specific to skeletal muscle myogenesis in myotubes while marker peaks for *Pax7*^*hi*^ clusters indicate that they have a supportive role during development, such as by providing structural integrity to myotubes through ECM remodeling. Interestingly, cluster A9 by itself displays terms that are related to neuromuscular junction formation such as axonogenesis (*P* = 0.0003) and generation of neurons (*P* = 0.002), which indicates that a subset of differentiated nuclei might play a specialized role compared to the rest of the differentiated population (Additional file 1: Fig. S6H).

### LR-Split-seq identifies differential TSS choice

We developed a peak calling script to identify TSSs and TESs from long-read data (Methods). For both bulk and single-cell data, reads filtered by known, NIC, NNC, and prefix ISMs for TSSs or suffix ISMs for TESs were scanned with a window of 50 bp to call TSS and TES peaks. Each end was required to be supported by at least 2 long reads (Additional file 1: Fig. S7A). We further filtered the ends at the level of each gene to achieve a refined set of TSSs and TESs for the bulk and LR-Split-seq data separately: 22,938 TSSs in bulk (Additional file 1: Fig. S7B, S7C, Additional file 2: Table S10), 23,996 TSSs in LR-Split-seq (Fig. 4B, Additional file 2: Table S8), 14,120 TESs in bulk (Additional file 1: Fig. S7D, S7E, Additional file 2: Table S11), and 12,521 TESs in LR-Split-seq (Additional file 1: Fig. S7F, S7G, Methods, Additional file 2: Table S9).

We performed the same complexity analysis on the identified TSSs and TESs per gene per cell that we did on the isoform level. We found nearly identical results where the cells and nuclei with more reads have a higher number of genes that express more than one TSS or TES and that the cells exhibit more TSS or TES complexity overall (Additional file 1: Fig. S7H). Comparing the number of distinct ends to the number of distinct splice isoforms revealed that multiple TSSs are expressed per single splice isoform in both bulk and single cells (Additional file 1: Fig. S7B, Fig. 4B). *Tnnt2* (troponin T2) has multiple known isoforms [39] and is differentially expressed between *Myog*^*hi*^ and *Pax7*^*hi*^ nuclei in the short-read data, so we decided to investigate chromatin accessibility and TSS usage at the *Tnnt2* locus (Additional file 1: Fig. S5D, Additional file 2: Table S5, Fig. 4C). We recovered four distinct TSSs for *Tnnt2*, three of which (Tnnt2_2, Tnnt2_3, and Tnnt2_4) overlap snATAC pseudobulk peaks, and all four of which overlap prior CAGE peaks found in C2C12 [40]. Tnnt2_4 overlaps a known promoter cCRE and GENCODE vM21 transcript start site, while Tnnt2_2 overlaps a distal enhancer cCRE (Fig. 4C) [41]. Tnnt2_4 has both higher expression in the LR-Split-seq data and increased accessibility in snATAC *Myog*^*hi*^ and *Pax7*^*hi*^ clusters, while Tnnt2_2 and Tnnt2_3 are more highly expressed and accessible in MB clusters (Additional file 1: Fig. S7I-J, Additional file 1: Fig. S8A). Therefore, an isoform switch occurs in *Tnnt2* where *Myog*^*hi*^ and *Pax7*^*hi*^ nuclei mainly use the known TSS belonging to the longer isoform, while the MB nuclei mainly use TSSs belonging to shorter isoforms.

Genome-wide, we validated our TSS calls using an extended set of data: our snATAC pseudobulk peaks, GENCODE vM21 TSSs, ENCODE cCREs (promoter and proximal enhancer) from mm10, and C2C12 CAGE peaks, and found that the majority of the TSSs identified from LR-Split-seq are validated by at least one of these five other datasets (Methods, Fig. 4D).

Using the same strategy we implemented to detect isoform-switching genes, we performed differential TSS usage tests on our LR-Split-seq data (Methods). We again subset our LR-Split-seq data into 0 h MB nuclei, 72 h *Pax7*^*hi*^ nuclei, and 72 h *Myog*^*hi*^ nuclei, and performed pairwise tests. In the MB vs. *Pax7*^*hi*^ comparison, we found 42 genes with differential TSS usage (Additional file 2: Table S12). In the MB vs. *Myog*^*hi*^ comparison, we found 40 genes with differential TSS usage. Consistent with our previous findings, this list includes *Tnnt2* (*Adj. P* = 6.16 × 10^−14^ MB vs. 72 h *Myog*^hi^), where the MB nuclei only express isoforms consistent with downstream TSSs (Tnnt2_1, Tnnt2_2, Tnnt2_3) (Additional file 2: Table S13). Conversely, the *Myog*^*hi*^ subset predominantly expresses isoforms using the upstream TSS (Tnnt2_4) (Fig. 4C, E).

Similarly, we found multiple distinct TESs per splice isoform in bulk and LR-Split-seq data (Additional file 1: Fig. S7D, S7F). We validated genome-wide TESs using GENCODE vM21 TESs and polyA-seq peaks from C2C12 cells at days 0 and 4 of differentiation, which overlapped the majority of TESs found in bulk data but not in the LR-Split-seq data (Additional file 1: Fig. S7E, S7G) (Methods). We believe that the apparent lack of external validation for the LR-Split-seq TESs is largely driven by the random priming method. When we call TESs instead using the same set of reads but split by priming strategy, 50.3% of the oligo-dT TESs validate by at least one form of external support compared to 6% of the random hexamer TESs (Additional file 1: Fig. S8B). When we use the same strategy to compare TSSs from oligo-dT-primed reads to those from random hexamer-primed reads, 83.5% of the oligo-dT TSSs validate by at least one form of external support compared to 84.8% of the random hexamer TSSs (Additional file 1: Fig. S8C, S8D).

We furthermore demonstrated the utility of LR-Split-seq for identifying TSSs and TESs by comparing how well our long-read ends are supported by external validation in comparison to those we called with from our companion short-read Split-seq data for the same cells. Only 50.2% of TSSs called using the short reads had external validation compared to 81.5% of LR-Split-seq TSSs (Fig. 4D, Additional file 1: Fig. S8E) (Methods). Similarly, the short-read Split-seq TESs validate externally at a much lower rate (12.3%) than the LR-Split-seq TESs (44.2%) (Additional file 1: Fig. S7G, Additional file 1: Fig. S8F) (Methods).

### Coordination of chromatin accessibility with transcriptional output

We calculated snATAC TSS chromatin accessibility across our refined set of TSSs to determine how TSS accessibility relates to TSS expression. We found that binary chromatin accessibility at a TSS was a particularly strong indicator of whether or not a TSS was expressed in MBs (65.7% of TSSs) (Additional file 1: Fig. S8G) and that the level of accessibility at each TSS correlated well with the expression level of each TSS (Pearson r = 0.44, Spearman rho = 0.58) (Methods). In the *Pax7hi* and *Myoghi* populations, accessibility at each TSS did not correlate as strongly (Pearson *r* = 0.58, Spearman rho = 0.20; Pearson *r* = 0.53, Spearman rho = 0.16 respectively). We then determined how many genes with more than one TSS displayed the highest accessibility level and expression level at the same TSS. In the myoblasts, the most highly accessible TSS for a gene was most often also the most highly expressed TSS for the gene (77.8% of genes with > 1 TSS). This concordance was less strong in the *Pax7hi* and *Myoghi* groups (54.6% and 52.7% respectively).

We then investigated which TSSs are supported by both differential accessibility and expression (Methods). We compared the average log2 fold change (LFC) in both accessibility and expression between *Myog*^*hi*^ and MB (Fig. 4F), *Pax7*^*hi*^ and MB (Fig. 4G), and *Myog*^*hi*^ and *Pax7*^*hi*^ (Fig. 4H). Between MB and *Myog*^*hi*^, 19 TSSs are specific to *Myog*^*hi*^ with an average LFC greater than two standard deviations (indicated by dashed lines) in both datasets, and 70 TSSs are specific to MB with average LFC less than two standard deviations in both datasets (Fig. 4F, Additional file 2: Table S14). Several of the genes with such TSSs are differentially expressed (Additional file 2: Table S5, Additional file 2: Table S14). Only 6 TSSs were *Pax7*^*hi*^-specific relative to MB, but one of these is *Igfbp5*, which is a gene that was highly differentially expressed in the *Pax7*^*hi*^ subset (Fig. 4G, Additional file 1: Fig. S5D, Additional file 2: Table S3, Additional file 2: Table S14). Comparing MB and *Pax7*^*hi*^, 77 TSSs are MB-specific, 36 of which are also MB-specific when comparing *Myog*^*hi*^ with MB. Of the 19 *Myog*^*hi*^-specific TSSs between *Myog*^*hi*^ and MB, 15 were also *Myog*^*hi*^-specific when compared to *Pax7*^*hi*^ (out of 53 total) (Fig. 4H, Additional file 2: Table S14). Several of the 17 *Pax7*^*hi*^-specific TSSs (Fig. 4H) belong to differentially expressed genes, such as *Pax7*, *Col4a1*, *Fn1*, and *Igfbp5* (Additional file 1: Fig. S5D, Additional file 2: Table S5, Additional file 2: Table S14). From a biological perspective, *Prox1* and *Vgll4* are potentially interesting; although they were not differentially expressed in the short-read data, they are known to be involved in skeletal muscle regeneration (Fig. 4H, Additional file 2: Table S14) [42, 43].

## Discussion

The first goal of this work was to advance our capacity to directly map and quantify RNA isoforms in single cells. Using the C2C12 myogenic differentiation as a test system, we introduce long-read Split-seq (LR-Split-seq) and show that it can be as effective as standard short-read Split-seq for detecting cell clusters, based on data from the same number of cells or nuclei. This conclusion applied to nuclei as well as whole cells, although whole-cell data detected more genes per cell than companion LR-Split-seq data from nuclei. For biological systems that do not permit uniform whole-cell disaggregation such as our multinucleated myotubes or brain tissue, the success shown here for nuclei is encouraging. We speculate that the remaining sensitivity differential between nuclei and whole cells is a consequence of the smaller starting number of transcripts in nuclei, and some of that could be further compensated by increasing the nuclear number sequenced and their depth of sequencing. We also suggest that combining random hexamer primed long reads with the oligo-dT primed long-read data helped to capture 5′ ends that are critical for inferring TSS use, although this adds incomplete PacBio reads to the overall dataset. We also illustrate that LR-Split-seq affords users the choice of analyzing the oligo-dT primed and hexamer primed read populations separately. A second motive for developing LR-Split-seq is that it will allow flexible study designs that can efficiently and more economically refine cell type identities by integrating additional standard short-read Split-seq data on the same samples. Results presented here showed that this strategy was effective in refining stem cell identities and states in the C2C12 system. Finally, we integrate results from LR-Split-seq with snATAC to gain insights into the dynamics of chromatin accessibility at the corresponding promoters with a longer-term goal of building a fully integrated model of physically or genetically affiliated distal regulatory elements.

We were able to detect 79% of the genes and 53% of transcript isoforms detected in bulk myoblast long-read RNA-seq using LR-Split-seq in single cells. We expect these differences relative to bulk samples to be a function of the individual study design, including number and diversity of cells sequenced, depth of sequencing, fixation protocol and, for isoform detection, and the contribution from internal hexamer priming. The largest sets of genes detected across the entire analysis included the LR-Split-seq assays, supporting the conclusion that it detects expressed genes reliably and reproducibly. The differences between known gene and transcript detection rates, relative to bulk data, were largely attributable to internally primed Split-seq reads and their management in our computation pipeline. Specifically, we used TALON, which leverages non-full-length reads for quantification and detection on the gene level but not on the transcript level. Consequently, we achieved high gene detection concordance but lower transcript detection concordance between long-read bulk and LR-Split-seq data.

Gene-level clusters in LR-Split-seq are remarkably similar to the results in the equivalent standard short-read Split-seq. In both assays, clusters of differentiating cells were most homologous to each other and were distinct from the myoblast clusters. However, in LR-Split-seq, there was a greater tendency for the clusters to separate by assay format, as shown in the 0 h myoblast cells and nuclei. We captured expression dynamics of well-known myogenic marker genes in the differentiating clusters such as *Pax7, Myog, Mybph,* and *Myh3* that are reproducible in the short-read data we sequenced from the same cells [18]. The additional context from ~ 37,000 short-read single cells allowed us to investigate the *Myog*^*hi*^ clusters in greater detail. We found that *Myog*^*hi*^ clusters were very distinct from MB clusters, while *Pax7*^*hi*^ clusters were in a spectrum of differentiation stages between MB and *Myog*^*hi*^ clusters. Expression of additional marker genes in *Pax7*^*hi*^ subclusters, RNA velocity trajectories, and validation with spatial transcriptomic profiling confirmed that these nuclei are from mononucleated cells in varying stages of differentiation.

LR-Split-seq enabled us to investigate transcript-level differences between the various stages of differentiation in myogenesis. We found novel insights into the biology of the system by studying differential TSS usage and integrating our TSSs identified from long reads and our snATAC-seq peaks. Our analysis revealed over 50 significant switches in TSS usage across clusters of undifferentiated versus differentiated stages, including a pronounced switch in *Tnnt2*, where the myoblasts primarily use TSSs that are novel to more recent GENCODE transcript annotations, while differentiated cells mainly express the known TSS that results in a longer isoform. This TSS switch was complemented by a corresponding increase in chromatin accessibility at the newly expressed TSS in *Myog*^*hi*^ clusters.

Unlike previous long-read scRNA-seq methods that rely on sequencing of each cell using custom microfluidics equipment [2, 4], LR-Split-seq is immediately accessible with no cell/droplet handling instrumentation and it is tunable in both cell number and sequencing depth, depending on the complexity of the underlying sample’s cellular composition. Additionally, it can be scaled up for long-read sequencing with additional sublibraries and higher read depth. We believe that this will allow one to optimize the amount and character of information from short- and long-read single-cell technologies when the costs of input cells, overall platform, and sequencing are all considered. While short-read Split-seq provides a broad survey of the transcriptional complexity of a biological system by sequencing up to 100,000 cells, corresponding LR-Split-seq can be applied to a targeted number of cells to provide higher-resolution isoform-level insights using a few million long reads from a few PacBio runs. In this way, LR-Split-seq promises relatively affordable, simultaneous transcriptional profiling of a wide variety of tissues using short- and long-read sequencing.

## Methods

### C2C12 culture and differentiation

C2C12 cells were purchased from the American Type Culture Collection (ATCC, CRL-1772). All cells used in experiments were passaged less than 10 times from the original plug. C2C12 were authenticated by testing for differentiation efficiency upon receipt. They were not tested for mycoplasma throughout the course of the study. C2C12 cells were cultured on 10-cm plates (Thermo Scientific, 172931) in 10 mL myoblast growth media: high-glucose DMEM with L-glutamine and without sodium pyruvate (HyClone, SH30022.FS), supplemented with 20% fetal bovine serum (Omega Scientific, FB-11), 100 units/mL penicillin, and 100 μg/mL streptomycin (Gibco, 15140122). Cells were maintained at 20–50% confluency at 37 °C with 5% CO_2_ and passaged at 1:3 or 1:4 every 2 to 3 days. To detach them from plates, cells were rinsed with 1× PBS (HyClone, SH30256.02) and incubated with 2 mL TrypLE-Express (Gibco, 12605010) for 5 min at 37 °C, which was then neutralized with 8 mL myoblast growth media. To differentiate, cells at 90–100% confluency were rinsed with 1× PBS and myoblast growth media was replaced with 10 mL differentiation media: high-glucose DMEM with L-glutamine and without sodium pyruvate (HyClone, SH30022.FS), supplemented with 2% donor horse serum (Gibco, 16050130), 100 units/mL penicillin, 100 μg/mL streptomycin (Gibco, 15140122), and freshly added 1 μM insulin (Sigma-Aldrich I6634). Differentiation media was replaced every 24 h for 3 days. Cells were monitored under a microscope (EVOS FL Auto 2) to observe changes in morphology and confirm differentiation.

### Preparation of myoblast and myotube single-nucleus suspensions

We followed the Bio-Rad SureCell WTA 3′ Library Prep protocol for preparation of nuclei samples [44]. Myoblasts from one 10-cm plate (~ 1.5 million cells) and myotubes from one 10-cm plate (~ 5 million cells) with > 90% viability were lifted as described above and pelleted in 15-mL polypropylene falcon tubes (VWR, 89039-670) by centrifuging for 5 min at 1500 RPM. Cells were washed twice with cold 1× PBS + 0.1% BSA (Sigma-Aldrich A9418) and 0 h myoblasts were filtered through a 40-μm strainer; due to their size, 72-h samples containing myotubes were not filtered. After centrifuging for 3 min at 300×*g*, cells were resuspended in 1 mL cold lysis buffer: 10 mM Tris-HCl pH 8 (Thermo Scientific, AM9855G), 10 mM NaCl (Fisher Scientific, S271), 3 mM MgCl_2_ (Sigma, M8266), 0.1% IGEPAL CA-630 (Thermo Scientific, 28324), 0.2 U/μL SUPERase In RNase Inhibitor (Invitrogen, AM2694), and 10 mg/mL BSA in nuclease-free water (Ambion, AM9937). Cells were incubated in lysis buffer on ice for 10 min, centrifuged at 4 °C for 3 min at 300×*g*, and washed with 1 ml of cold 1× PBS + 1% DEPC water (Invitrogen, 750023). The lysis, spin, and wash steps were repeated two more times for the 72 h samples because myotube cell membranes are more difficult to fully lyse than mononucleated myoblasts. Nuclei were stained with Trypan Blue (Bio-Rad, 1450021), and cell membrane lysis was confirmed under a microscope and by percent viability (< 10%). Nuclei were stored on ice in 1 mL nuclei storage buffer (lysis buffer without the addition of IGEPAL CA-630).

### Preparation of single-cell barcoded cDNA using Split-seq

Single-cell barcoded cDNA and Illumina libraries were prepared using the Fixation Kit for Cells, Fixation Kit for Nuclei, and Single Cell Whole Transcriptome Kit (Parse Biosciences, SB2001) following the manufacturer’s protocols. Nuclei from the 0-h myoblast sample and 72-h sample in single-nucleus suspensions were counted on a TC20 Automated Cell Counter (Bio-Rad, 1450102), and ~ 4 million were filtered through a 40-μm strainer into 15-mL polypropylene falcon tubes. Nuclei were fixed for 10 min and permeabilized for 3 min on ice, then DMSO was added for storage overnight at − 80 °C in a Mr. Frosty. Myoblast cells were similarly counted and filtered through a 40-μm strainer, followed by fixation and permeabilization. DMSO was added and cells were stored overnight at − 80 °C in a Mr. Frosty. Before storage, single-cell and single-nucleus suspensions were confirmed under a microscope.

To prepare barcoded cDNA, fixed and frozen cells and nuclei were thawed in a 37 °C water bath and counted. Cells were added to the Round 1 reverse transcription barcoding plate at around ~ 15,000 cells/well, with A1-A12 containing 0 h cells, B1-B12 containing 0 h nuclei, and C1-D12 containing 72 h nuclei (Additional file 1: Fig. S2A), before in situ reverse transcription and annealing of barcode 1 + linker on a thermocycler (Bio-Rad T100). After RT, cells were pooled using a multichannel pipette into a 15-mL tube, spun down at 4 °C for 5 min at 1000×*g*, and resuspended in 1 mL of Resuspension Buffer (Parse Biosciences, SB2001). Using a basin and multichannel pipette, cells were distributed in 96 wells of the Round 2 ligation barcoding plate for the in situ barcode 2 + linker ligation. Next, cells were pooled, filtered through a 40-μm strainer, and redistributed into 96 wells of the Round 3 ligation barcoding plate for the in situ barcode 3 + UMI + Illumina adapter ligation. After a final pooling and filtration through a 40-μm strainer, cells were counted using a hemocytometer and distributed into 7 sublibraries: 6 sublibraries with 9000 cells each, and 1 sublibrary with 1000 cells. The cells in each sublibrary were lysed and libraries were cleaned with AMPure XP beads (Beckman Coulter, A63881), then the single-cell barcoded cDNA underwent template switching and amplification. Importantly, we increased the number of cycles for the 1000-cell library to 20 cycles rather than 18 in order to increase the yield of single-cell barcoded cDNA for use in Illumina library preparation (50 ng) while having enough leftover cDNA for PacBio library preparation (500 ng). The cDNA was cleaned using AMPure XP beads and quality checked using an Agilent Bioanalyzer before proceeding to Illumina and PacBio library preparation.

### Preparation of Illumina scRNA-seq libraries using Split-seq and sequencing

All 7 sublibraries were fragmented, size-selected using AMPure XP beads, and Illumina adapters were ligated. The cDNA fragments were cleaned again using beads and amplified, adding the fourth barcode and P5/P7 adapters, followed by a final bead-based size selection and quality check with a Bioanalyzer. Libraries with 5% PhiX spike-in were loaded at 2.1 pM and sequenced to an average depth of 51 million reads per 9000-cell library and 70 million reads for the 1000 cell library using an Illumina NextSeq 500 with paired-end run configuration 74/86/6/0. The data are hosted on GEO (GSE168776) and on the ENCODE portal (ENCBS521YWL, 0 hr cells, ENCBS431NOZ, 0hr nuclei; and ENCBS978ZNQ, 72hr nuclei).

### Preparation of PacBio scRNA-seq library and sequencing

The PacBio library was prepared using 500 ng of amplified, single-cell barcoded cDNA with the SMRTbell Template Prep Kit (PacBio, 100-938-900) according to the manufacturer’s protocol for sequencing on a Sequel II. The 1000-cell library was sequenced using 2 SMRTcells (PacBio, 101-008-000) for a sequencing depth of 5,764,421 full-length non-chimeric reads. The data are hosted on GEO (GSE168776) and on the ENCODE portal (ENCBS521YWL, 0hr cells, ENCBS431NOZ, 0hr nuclei; and ENCBS978ZNQ, 72hr nuclei).

### Preparation of bulk PacBio libraries and sequencing

We extracted RNA from two replicates of C2C12 0 h samples and 72 h samples using the RNA-easy kit (Qiagen, 74104). cDNA synthesis and library preparation using the SMRTbell Template Prep Kit (PacBio, 100-938-900) were performed as described on the ENCODE portal (https://www.encodeproject.org/documents/77db752f-abf7-4c93-a460-510464134f52). We sequenced one SMRT cell per replicate on the Sequel II platform. The data are hosted on the ENCODE portal (ENCBS824FPY, ENCBS649CMC for 0hr cells; and ENCBS373BHL, ENCBS606QKU for 72hr cells).

### Preparation of snATAC-seq libraries using Bio-Rad technology and sequencing

The single nucleus ATAC-seq experiment was performed using the SureCell ATAC-Seq Library Prep Kit (Bio-Rad, 17004620) following the manufacturer’s protocol for the OMNI-ATAC version [45]. Cells at 0 h differentiation or 72 h differentiation timepoints in one 10-cm plate per biological replicate were lifted as previously described and washed twice in cold 1× PBS + 0.1% BSA. All 0 h replicates and some 72 h replicates were filtered through a 40-μm strainer (2 technical replicates, 1 biological replicate; 2 technical replicates of 72 h samples were not filtered), then counted and assessed for viability. A total of 300,000 cells with > 90% viability per biological replicate were lysed with cold OMNI-ATAC lysis buffer on ice for 3 min and washed out with cold ATAC-Tween buffer, at which point non-filtered 72 h nuclei were filtered through a 40-μm strainer, then spun down at 500 RCF for 10 min at 4 °C. Nuclei were resuspended, counted, and confirmed to be single-nucleus suspensions under a microscope, then 60,000 nuclei per biological replicate were tagmented at 37 °C for 30 min in a ThermoMixer with 500 RPM mixing. The microfluidics-based ddSEQ Single-Cell Isolator was used to stream tagmented nuclei in an amplification reaction mix with barcoded beads to isolate single nuclei in nanodroplets with one or more barcodes. Tagmented cDNA was barcoded and amplified, then nanodroplets were broken and libraries cleaned with AMPure XP beads before a second amplification of barcoded fragments and final bead-based cleanup. A Bioanalyzer was used to verify library quality before loading at 1.5 pM and sequencing to an average depth of 122 million reads per library using an Illumina NextSeq 500 with paired-end run configuration 118/40/8/0 and custom sequencing primer. The data are hosted on GEO (GSE168776) and on the ENCODE portal (ENCBS081AJF, ENCBS562OEW, 0hr nuclei; ENCBS779SXF, ENCBS143VME, 72hr nuclei; and ENCBS247OBN, ENCBS090IYH, 72hr nuclei isolated from filtered cells).

### Validation of transcript expression with RNAscope

Myoblasts were grown to 90–100% confluency in flasks mounted on slides (Thermo Scientific, 170920) then differentiated over 3 days as previously described. The flasks were removed and slides were rinsed in 1× PBS, followed by fixation in 10% neutral buffered formalin (Sigma-Aldrich, HT501128) for 30 min at room temperature. Following the manufacturer’s protocol for cultured adherent cells, we rinsed the slides in 1× PBS, then incubated in 50%, 70%, and 100% ethanol for 5 min each [46]. Slides were stored submerged in 100% ethanol at − 20 °C in 50-mL falcon tubes. To rehydrate, slides were incubated in 70% and 50% ethanol for 2 min each, then in 1× PBS for 10 min. A hydrophobic barrier was drawn around the edges of the slide (Vector Laboratories, H-4000), then the cells were permeabilized with 1:15 diluted protease III (ACDBio, 322340) for 10 min at room temperature in a humidity control tray (ACDBio, 310012). Following the manufacturer’s protocol for the RNAscope HiPlex12 kit (ACDBio, 324100/324140), probes for genes of interest were mixed and hybridized for 2 h at 40 °C in a HybEZ II hybridization oven (ACDBio, 321710/321720), then the signal was amplified over 3 rounds of 30 min incubations at 40 °C in the oven [47]. We then proceeded to fluorophore hybridization and imaging over four rounds of three channels per round (GFP, RFP, and Cy5) plus DAPI [48]. An EVOS FL Auto 2 with programmable stage was used to automatically image slides at × 40 magnification.

### Preprocessing of LR-Split-seq data

Raw PacBio reads were processed into circular consensus reads using the ccs software from the SMRT analysis software suite (parameters: --skip-polish --min-length = 10 --min-passes = 3 --min-rq = 0.9 --min-snr = 2.5) (https://github.com/PacificBiosciences/ccs). The Split-seq adapters were identified and removed using Lima (v2.0.0) (parameters: --ccs --min-score 0 --min-end-score 0 --min-signal-increase 0 --min-score-lead 0) (https://github.com/pacificbiosciences/barcoding/). Reads were then processed with IsoSeq3’s Refine (v3.4.0) to yield full-length non-chimeric reads (https://github.com/PacificBiosciences/IsoSeq). As around half of our reads are primed using random hexamer priming, polyA tails were not required nor removed for this step.

Reads were then demultiplexed for their Split-seq barcodes using a custom script (https://github.com/fairliereese/LR-splitpipe) by first detecting the spacer sequences between barcodes and using these as start and end points for the barcodes. Barcodes were corrected to those that were within an edit distance of 3 of the predetermined list of barcodes used for each round of barcoding. The resultant reads were then filtered on which combinations of barcodes were also seen in the Illumina single-cell/nucleus RNA-seq data, which yielded 567 of the 568 cells that passed QC in the Illumina data (Additional file 1: Fig. S2B). The reads were then trimmed of their barcodes to facilitate mapping, and cell identity barcodes were recorded. The reads were mapped using Minimap2 (v2.17-r94) (-ax splice:hq -uf --MD) [49] and the mm10 reference mouse genome, corrected for long-read sequencing artifacts with TranscriptClean ( --canonOnly --primaryOnly) [50]. We then used TALON (development branch on GitHub) (--cb) to annotate each read to its transcript or origin using the GENCODE vM21 reference (24). We filtered for reproducible novel NIC and NNC transcript models for those that were seen in 4 or more sub-cells (Fig. 1K, Additional file 1: Fig. S1D). Custom LR-Split-seq demultiplexer can be found at https://github.com/fairliereese/LR-splitpipe [51] or on Zenodo at 10.5281/zenodo.5168057. Figure generation code can be found at https://github.com/fairliereese/2021_c2c12 [52] or on Zenodo at 10.5281/zenodo.5168059. All code is available under the MIT open source license.

### Comparing priming strategies and sample types in LR-Split-seq data

The priming strategy of each read was determined by examining the barcode for the first round of Split-seq. Reads were separated out by priming strategy and by cell. For sample comparisons, the oligo-dT and random hexamer primed reads from each cell were merged to create the final cell, then separated out by sample.

### Comparing bulk long-read to LR-Split-seq

To enable this comparison, we re-ran the bulk and single-cell data through TALON using the same database so that novel transcripts would have the same IDs across the bulk and the single cell. For the bulk novel transcript models, filtering was done using talon_filter_trasncripts, requiring a novel transcript model to be reproducible in at least 2 of the bulk replicates with at least 5 copies. For the single cell, filtering was done that required novel transcript models to be reproducible in at least 4 sub-cells.

### Single-cell processing of LR-Split-seq data

Oligo-dT and random hexamer primed reads from each cell were merged to create the final cells. Gene-level cells and nuclei were further filtered for those that had ≥ 500 reads per cell/nucleus using Scanpy (v1.4.6) [53] and for those that, in the corresponding Illumina data, had < 200,000 reads, < 20% mitochondrial reads, and > 500 genes (done in Seurat as detailed in the Processing of short-read scRNA-seq data section) (all on a per cell/nucleus basis), yielding a final total of 464 single cells and nuclei. Dimensionality reduction, construction of the UMAP, and Leiden clustering were all performed using Scanpy, yielding 7 clusters (Fig. 2D).

### Isoform-switching gene testing

Testing for isoform switching in LR-Split-seq data was performed as in Joglekar et al. [4]. For each pairwise test, an *n* × 2 contingency table was created with counts in each condition for each isoform in a gene, with a maximum of 11 isoforms. In cases where a gene had more than 11 isoforms, an 11th entry was constructed where counts were summed for the most lowly expressed isoforms. Each gene was required to have at least 10 supporting reads from each condition to be considered testable. For each testable gene, a chi-squared test was performed and, Δπ., or the change in percent isoform usage for the gene, was computed as the sum of the absolute value of percent isoform usage across the conditions for the top two expressed isoforms. *P* values from the chi-squared test were corrected using Benjamini-Hochberg correction. Tests were performed on the LR-Split-seq Leiden clusters for MB nuclei vs. *Myog*^*hi*^ nuclei (clusters LR1-LR2 vs. LR6-LR7) and for MB nuclei vs. *Pax7*^*hi*^ (clusters LR1-LR2 vs. LR4-LR5). Genes with significant isoform switching were required to have a corrected *p* value ≤ 0.05, Δπ of ≥ 10, and a minimum number of reads per gene per tested condition of 10.

### Processing bulk long-read data

Bulk PacBio data was processed following the ENCODE Long-Read RNA-Seq Analysis Protocol for Mouse Samples (v.1.0) for CCS, Lima, refine and TranscriptClean steps (https://www.encodeproject.org/documents/a84b4146-9e2d-4121-8c0c-1b6957a13fbf). A TALON database was initialized using mm10 GENCODE v21 GTF with SIRV set 3 and ERCCs included. Reads output from TranscriptClean were labeled with the corresponding fasta reference. TALON was run (--cov 0.9 --identity 0.8). Filtering novel transcript models was done using TALON’s talon_filter_transcripts module, requiring a novel transcript model to be reproducible across biological replicates, and appear 5 times in each replicate, as well as display a lack of internal priming evidence (--minCount 5 --minDatasets 2 --maxFracA 0.5). Transcript abundances were determined using talon_abundance.

### Processing of short-read Split-seq data

After initial demultiplexing of the 7 sublibraries (6 × 9000-cell sublibraries and 1 1000-cell sublibrary), Parse Bioscience’s split pipe v0.7.6 software was used to deconvolute reads into single cells, map to mm10 using STAR (v. 2.6.0c), annotate using GENCODE vM21, and filter using a UMI cutoff determined by knee plots (Additional file 1: Fig.S2B, S2D) [54]. The remaining cells were further filtered in Seurat (v. 3.2.2) by < 20% mitochondrial reads, < 200,000 counts, and > 500 genes per cell/nucleus (Additional file 1: Fig. S2C, S2E) [55]. The resulting 464 cells with both short and long reads and 36,405 cells with short-read data only were analyzed using Velocyto (v.0.1.17) [25]. Fifty-five percent of counts from 0 h cells, 46% of counts from 0 h nuclei, and 37% of counts from 72 h nuclei were spliced out of the total number of spliced and unspliced counts. After loading the loom file back into Seurat with the ReadVelocity function from the SeuratWrappers package, SCTransform (v. 0.3.1) was used to regress percent mitochondrial reads, number of genes, and sublibrary, followed by UMAP dimensionality reduction [56, 57]. Clustering using the Leiden algorithm (v. 0.8.0) resulted in 20 clusters [58]. Differentially expressed genes per cluster were found using Seurat’s FindAllMarkers function (only.pos = TRUE, min.pct = 0.1, logfc.threshold = 0.1) then further filtered by FDR < 0.01.

### Processing of snATAC-seq data

After demultiplexing the 8 snATAC-seq libraries (3 0 h, 5 72 h samples), Bio-Rad’s dockerized ATAC-seq analysis toolkit (v.1.0.0) was used to recover barcodes/UMIs, align reads with BWA, filter and deconvolute barcodes, perform quality control by UMI thresholding, and call peaks with MACS2 (Additional file 1: Fig. S6A) [59–61]. A custom script (https://github.com/fairliereese/lab_pipelines/tree/master/sc_atac_pipeline) that takes in the combined peaks file, QC-passing barcode list, and mapped reads was used to generate peaks-by-cells counts matrices as csv files for each library. In addition, the annotated bam files were converted to fragment files using scATAC-pro’s simply_bam2frags.pl script, which are bed-like matrices containing chromosome, start, stop, cell ID, and number of fragments contained in the region [62]. Further QC cutoffs consisted of a TSS enrichment score > 6, > 5000 counts, and < 20,000 counts per nucleus (Additional file 1: Fig. S6B). TSS enrichment is calculated in Signac (v. 1.0.9004) following the definition by ENCODE (https://www.encodeproject.org/data-standards/terms/). Signac was used to normalize binarized peaks-by-cells count matrices by term frequency inverse document frequency (TF-IDF) followed by singular value decomposition and UMAP dimensionality reduction [37]. The Leiden algorithm (v. 0.8.0) was used to resolve 18 clusters (Fig. 4A). UCSC Genome Browser tracks were generated by splitting the snATAC bam file by cluster using the sinto package (https://github.com/timoast/sinto) and creating bigwig tracks using deeptools [63]. Differentially accessible peaks per cluster were found using Seurat’s FindAllMarkers function (only.pos = TRUE, min.pct = 0.1, logfc.threshold = 0.5) then further filtered by FDR < 0.05. The marker peaks were grouped by MB (A1-A7), *Pax7*^hi^ (A8-A15), and *Myog*^*hi*^ (A16-A17) and processed using GREAT with mm10 whole genome background and associating peaks with the single nearest gene within 50 kb [64]. *P* values for the binomial test are reported in the text. The resulting genes for cluster A9 were also input into Enrichr to determine enriched GO Biological Processes [65]. The clustergram was downloaded from the Enrichr web tool (https://maayanlab.cloud/Enrichr/).

### Integration of short-read Split-seq and snATAC-seq data

Signac’s FindTransferAnchors function was implemented with all 36,869 Split-seq cells as the reference set and all 23,525 snATAC-seq nuclei as the query set, with canonical correlation analysis (CCA) used as the dimensional reduction method [38]. The TransferData function was used to carry over Split-seq labels “0 h” or “72 h” in one analysis (Additional file 1: Fig. S6D, left panel) and labels “MB,” “*Pax7*^*hi*^,” and “*Myog*^*hi*^,” in another analysis (Additional file 1: Fig. S6D, right panel).

### Identification of TSSs from long-read data

For both LR-Split-seq and bulk separately, bam reads were filtered for those that were annotated by TALON as belonging to the known, novel in catalog (NIC), novel not in catalog (NNC), and prefix-ISM novelty categories as the starts of reads belonging to these novelty categories are more likely to come from a true 5′ end. TSSs were called on the filtered bams using the ENCODE PacBio TSS caller (https://github.com/ENCODE-AWG/tss-annotation/blob/master/long_read/pacbio_to_tss.py) (--window-size = 50 --raw-counts --expression-threshold = 0), yielding a bed entry for each TSS consisting of a wide peak, narrow peak, and a summit for each TSS. Resultant Split-seq TSSs were filtered first by requiring each one to be supported by at least 2 reads, and subsequently on the gene level, where each called TSS was required to have a number of reads > 10% of the number of reads that supported the most highly expressed TSS for the same gene. Bulk TSSs were similarly filtered except using a threshold of > 5%. For the oligo-dT versus random hexamer priming comparison, the aforementioned filtered reads were split based on their priming strategy before running the TSS caller. The intersection of oligo-dT TSSs and random hexamer TSSs was determined using bedtools v. 2.30.0 [66].

### Identification of TESs from long-read data

Similarly, for both LR-Split-seq and bulk data separately, bam reads were filtered for those annotated as belonging to the known, novel in catalog (NIC), novel not in catalog (NNC), and suffix-ISM novelty categories as the ends of reads belonging to these novelty categories are more likely to come from a true 3′ end. TESs were called on the filtered bam using the same ENCODE PacBio TSS caller (https://github.com/ENCODE-AWG/tss-annotation/blob/master/long_read/pacbio_to_tss.py) (--window-size = 50 --raw-counts --expression-threshold = 0 --tes), yielding a bed file the same format as the TSS file. The TESs were then filtered by requiring each to be supported by at least 2 reads and have a number of reads > 80% of the number of reads that supported the most highly expressed TES for the same gene. For the oligo-dT versus random hexamer priming comparison, the aforementioned filtered reads were split based on their priming strategy before running the TSS caller.

### Identification of TSSs from short-read data

Bam reads from the short-read Split-seq 464 cells were queried for those that contained the complete template-switching oligo sequence with no errors using Cutadapt v. 2.10 (-g AACGCAGAGTGAATGGG -e 0 -O 17), which represent reads that are more likely to contain a true 5′ end [67]. TSSs were called on the filtered bams using the ENCODE PacBio TSS caller (which despite its name works on short reads as well) https://github.com/ENCODE-AWG/tss-annotation/blob/master/long_read/pacbio_to_tss.py) (--window-size = 50 --raw-counts --expression-threshold = 0), yielding a bed entry for each TSS consisting of a wide peak, narrow peak, and a summit for each TSS. Resultant TSSs were filtered by requiring each one to be supported by at least 20 reads.

### Identification of TESs from short-read data

Bam reads from the short-read Split-seq 464 cells were queried for those that contained a run of 20 bp where at least 10 bases were “A”s Cutadapt v. 2.10 (-g AAAAAAAAAAAAAAAAAAAA -e 0.5 -O 20), which represent reads that are more likely to contain a true 3′ end [67]. TESs were called on the filtered bams using the ENCODE PacBio TSS caller (which despite its name works on short reads as well (https://github.com/ENCODE-AWG/tss-annotation/blob/master/long_read/pacbio_to_tss.py) (--window-size = 50 --raw-counts --expression-threshold = 0 --tes), yielding a bed entry for each TES consisting of a wide peak, narrow peak, and a summit for each TES. Resultant TESs were filtered by requiring each one to be supported by at least 20 reads.

### Processing C2C12 CAGE data

CAGE data was downloaded from GEO accession GSE21580 [40, 68]. Wig files corresponding to CAGE data from days 0 and 9 of C2C12 differentiation were converted to bed format using bedops wig2bed [69] and lifted over from the mm9 genome to the mm10 genome using UCSC’s liftOver tool (-minMatch = 0.95) [65]. Resultant bed peaks were concatenated.

### Processing C2C12 PolyA-seq data

PolyA-seq data was downloaded from GEO accession GSE62001 [70, 71]. Entries in the provided expression matrix were filtered for those belonging to the “C2C12.Pro” (proliferating C2C12) and “C2C12.Diff” (4-day differentiation C2C12) categories. The data was then converted into bed format using a custom script and lifted over from mm9 to mm10 using the UCSC liftOver tool (-minMatch = 0.95) [72].

### Intersecting TSSs with validation datasets

A combined TSS validation bed file was made using the proximal enhancer and promoter ENCODE cCREs [41], GENCODE vM21 TSSs [73], our snATAC-seq pseudobulk peaks, and CAGE peaks [40]. The filtered TSSs for both bulk (22,938) and LR-Split-seq (23,996) were intersected with the combination bed file using bedtools intersect 2.30.0 with default parameters, meaning minimum of 1 bp overlap between the TSSs and the combined validation set (2,057,291 regions) [66].

### Intersecting TESs with validation datasets

A combined TES validation bed file was made using our snATAC-seq pseudobulk peaks and polyA-seq peaks [70]. Similar to TSS validation, bedtools intersect with default overlap settings (1 bp) was used to determine the number of overlaps between our filtered TESs for both bulk (14,120) and LR-Split-seq (12,521) and the combined validation set (205,853 regions).

### snATAC-seq and TSS integration

TSS regions identified in LR-Split-seq in bed format were used to calculate activity at each TSS through the Signac interface. Normalized expression values and normalized TSS activity values were averaged across the three groups of cells (MB, *Pax7*^*hi*^, and *Myog*^*hi*^) and a pseudocount of 1 was added to each TSS. Fold change in expression and activity separately was calculated by dividing the TSS values of one group by another group, such as *Myog*^hi^/MB. The log2 fold change for each TSS was then plotted for both expression (*x*-axis) and activity (*y*-axis), revealing TSSs with chromatin profiles and expression in agreement at the upper right and bottom left sectors. Twice the standard deviation of each dataset is indicated by black dashed lines (Fig. 4F–H). We determined whether a TSS was expressed and/or accessible using a read cutoff of 2 for LR-Split-seq data and a cutoff of 1000 for normalized snATAC-seq data (Additional file 1: Fig. S8G).

### LR-Split-seq TSS quantification and differential TSS testing

TSS expression was quantified from the LR-Split-seq data starting from the TALON read annotation file (Additional file 2: Table S1), which tracks the start and end coordinates of every read. Read starts were converted to a read start bed file and expanded to include ± 25 bp from the true start. Finally, the read start bed was intersected using bedtools with the filtered LR-Split-seq TSSs (Additional file 2: Table S8), requiring at least 1 bp of overlap. The number of reads per TSS was then computed by counting all of the reads assigned to each TSS. Testing on the TSS level for the LR-Split-seq data was performed as in Joglekar et al. [4]. Tests were performed on the LR-Split-seq Leiden clusters for MB nuclei vs. *Myog*^*hi*^ nuclei (clusters LR1-LR2 vs. LR6-LR7) and for MB nuclei vs. *Pax7*^*hi*^ (clusters LR1-LR2 vs. LR4-LR5). Genes with significant TSS switching were required to have a corrected *p* val ≤ 0.05 and a change in percent isoform usage per condition of ≥ 10, and a minimum number of reads per gene per tested condition of 10. A custom UCSC Track Hub displaying pseudobulk snATAC peaks per cluster, LR-Split-seq reads used for TSS calling per cluster, ENCODE cCREs, and GENCODE vM21 transcript models can be found at https://github.com/erebboah/c2c12_trackhub. An interactive web app for viewing C2C12 Split-seq data can be found at http://hubble.bio.uci.edu.

## Supplementary Information

**Additional file 1:.** Fig. S1-S8.

**Additional file 2:.** Tables S1-S14.

**Additional file 3:.** Review history
