# Supplementary material for: Mapping and modeling the genomic basis of differential RNA isoform expression at single-cell resolution with LR-Split-seq
Source: Genome Biol. 2021 Oct 7;22:286. doi: 10.1186/s13059-021-02505-w (PMC8495978; doi:10.1186/s13059-021-02505-w)
Supplement: Supplementary file 1 — Additional file 1:. Fig. S1-S8. [file 13059_2021_2505_MOESM1_ESM.pdf]

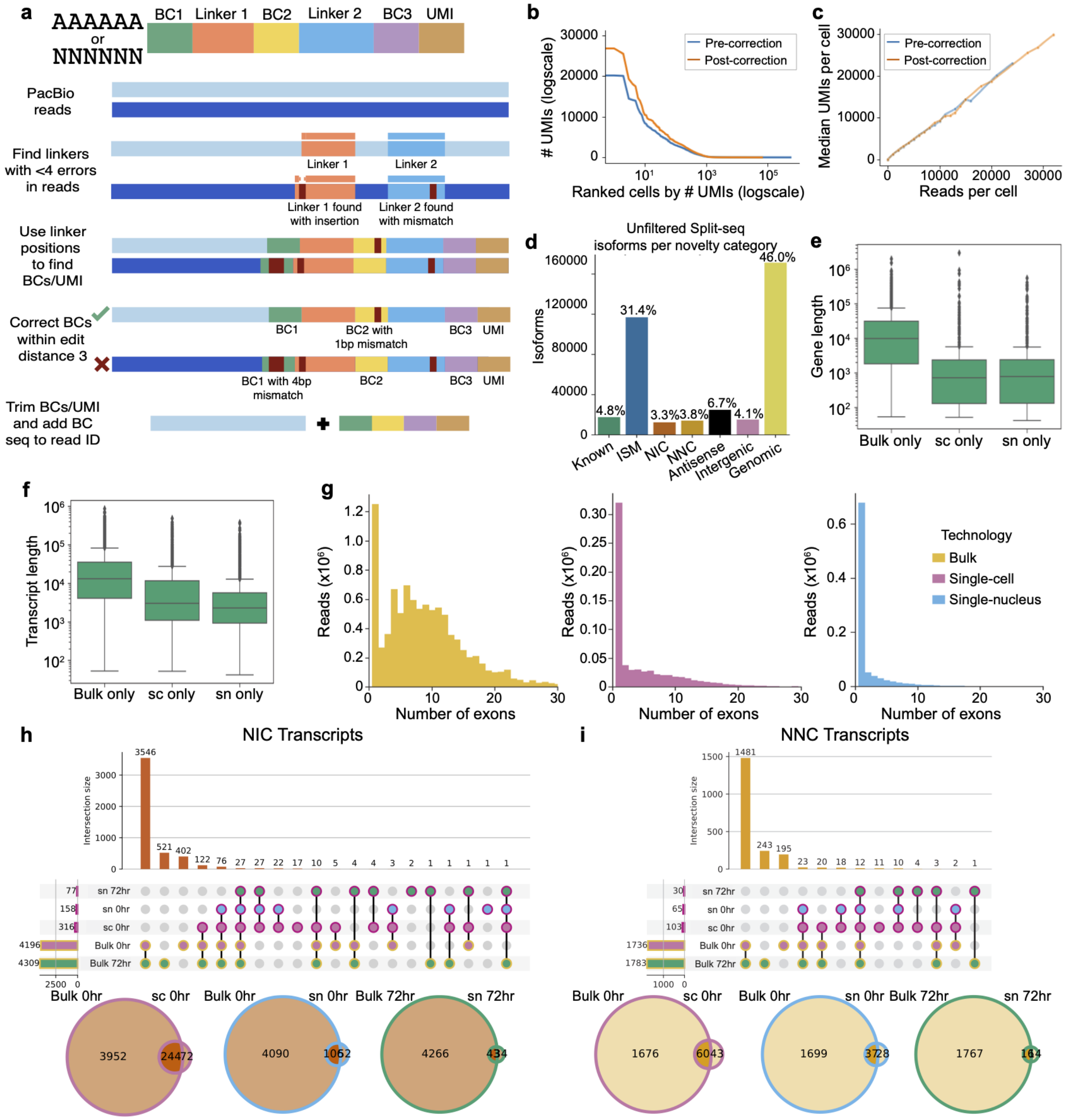

Fig. S1: LR-Split-seq preprocessing, QC, and additional analysis. a, Schematic diagram of LR-Split-seq demultiplexing strategy. **b**, UMI per ranked barcode plots before and after barcode correction (both axes log scaled). **c**, Median number of UMIs per cell binned by reads per cell before and after barcode correction. **d**, Unfiltered isoforms per novelty category across all cells in LR-Split-seq data. **e**, Gene lengths of annotated genes detected in bulk only, single-cell only, and single-nucleus only (log scale). **f**, Transcript lengths of annotated transcripts detected in bulk only, single-cell only, and single-nucleus only (log scale). **g**, Distribution of number of exons in bulk long reads (yellow), single-cell long reads (pink), and single-nucleus long reads (blue). **h**, Upset plot of novel in catalog (NIC) transcripts that passed filtering found in bulk data

compared to single cell data across all samples. Bars on the left indicate set size, circles indicate various combinations of samples, and bars on top indicate the number of genes found in each combination. Outline colors indicate technology (bulk in yellow, single-cell in magenta) and fill colors indicate sample type (72hr nuclei in green, 0hr nuclei in blue, and 0hr cells in pink for single-cell data; 72hr in green, 0hr in pink for bulk data). Box plots above indicate gene length distribution for each intersection. Venn diagrams below summarize the overlaps between bulk (left) and single-cell or single-nucleus (right), for each sample type. Sample type is indicated by outline color. **i**, Upset plot and Venn diagrams of novel not in catalog (NNC) transcripts that passed filtering found in bulk data and single-cell data.

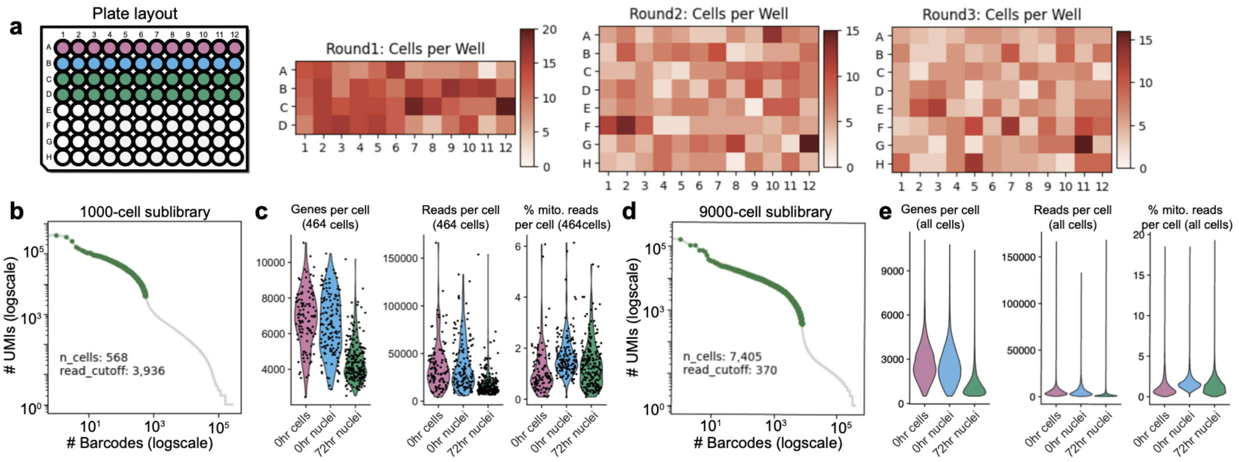

Fig. S2: Short-read Split-seq QC. a, Schematic of sample type per well in the first round of barcoding (pink = 0hr cells, blue = 0hr nuclei, and green = 72hr nuclei). Panels to the right show the number of cells per well across each round of barcoding for a 9,000-cell sublibrary. **b**, UMI per cell knee plots for the 1,000-cell sublibrary sequenced with both long and short reads indicating a threshold of 3,936 reads per cell, leaving 568 cells before additional QC. **c**, Violin plots of scRNA-seq QC metrics after filtering for the 464 cells only. **d**, An example knee plot for a 9,000-cell sublibrary indicating a threshold of 370 reads per cell, leaving 7,405 cells before additional QC. **e**, Violin plots of scRNA-seq QC metrics after filtering for all cells.

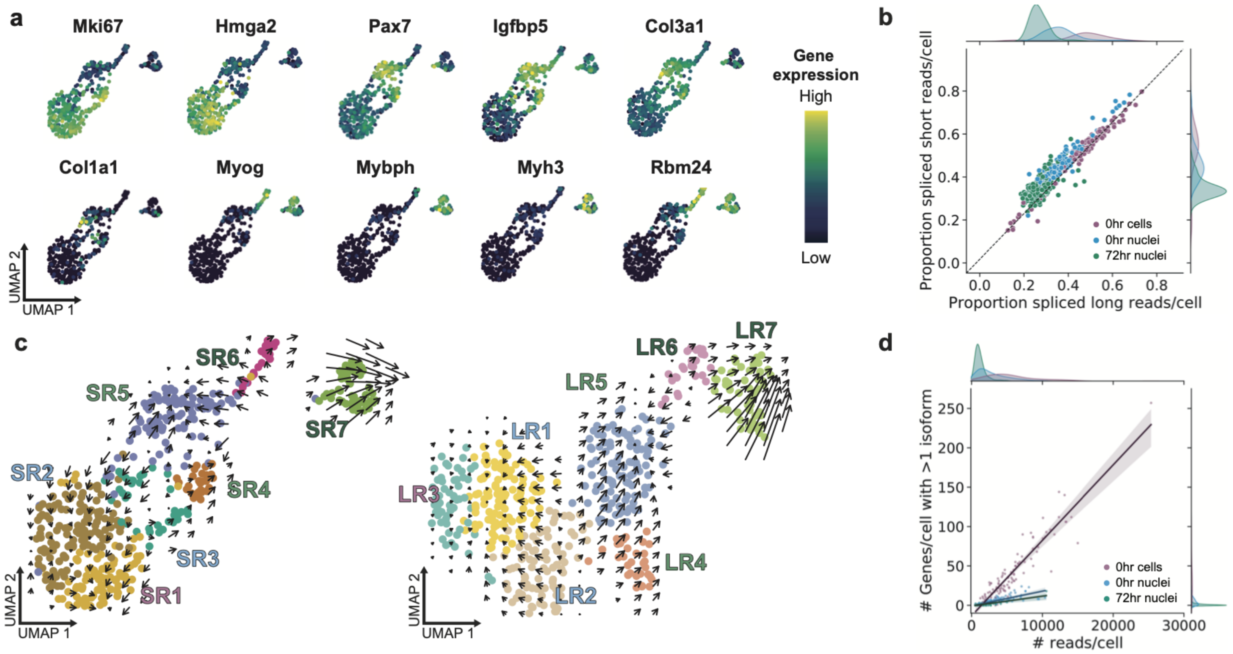

Fig. S3: Short-read and LR-Split-seq additional analysis. a, Distribution of marker genes within the 464-cell UMAP (dark blue = lowly expressed, yellow = highly expressed). **b**, Proportion of spliced vs. unspliced reads per cell in short-read Split-seq and LR-Split-seq from RNA velocity analysis. Cells are labeled by sample type (0hr cells in pink, 0hr nuclei in blue, and 72hr nuclei in green) and marginals on the top and right indicate their distributions **c**, Short-read (left) and LR-Split-seq (right) UMAPs for 464 cells with RNA velocity field trajectories indicated by arrows. **d**, Isoform complexity (Number of genes with more than one isoform per cell) vs. number of reads per cell, colored by sample type (0hr cells in pink, 0hr nuclei in blue, and 72hr nuclei in green).

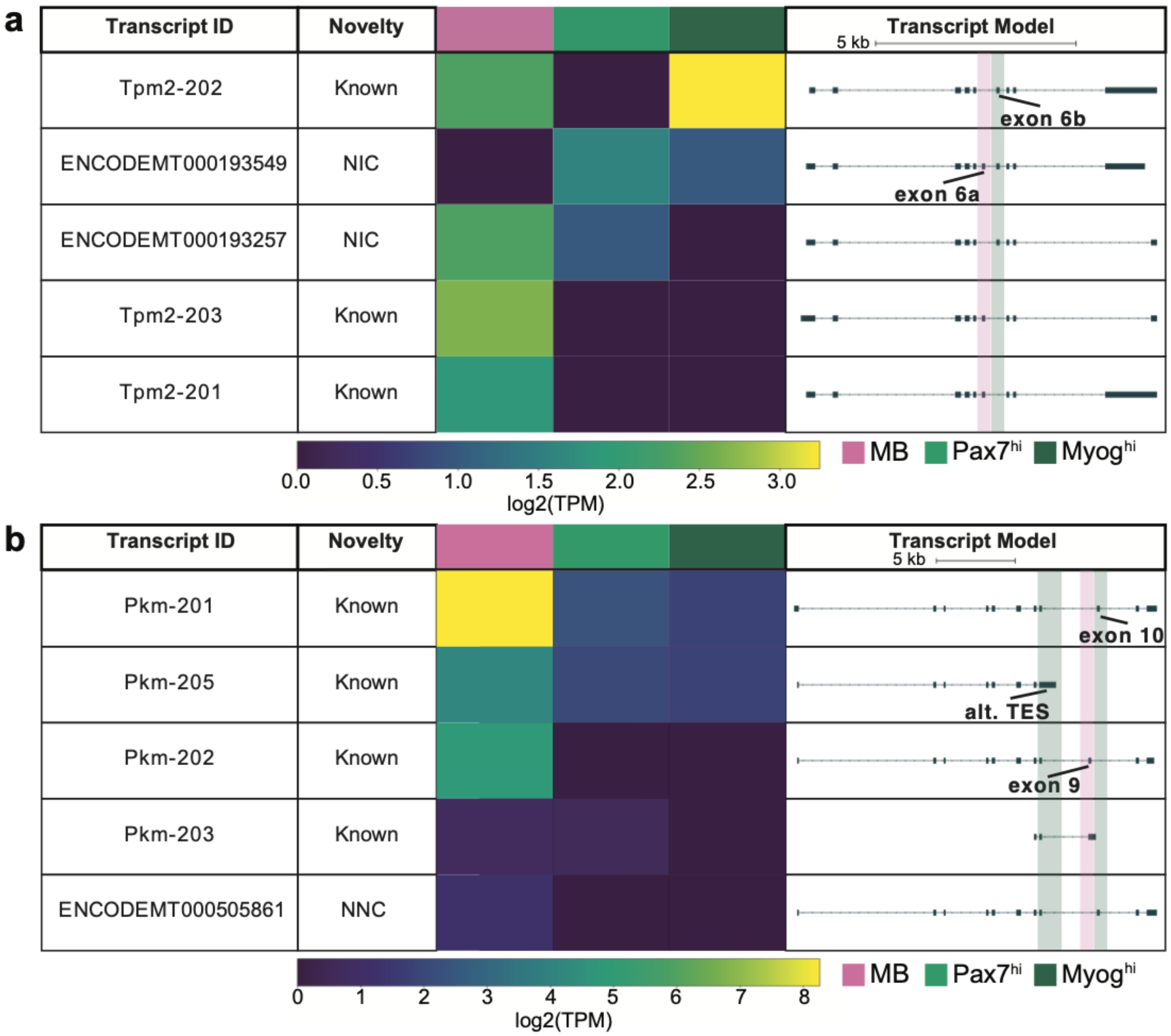

Fig. S4: Swan analysis of *Tpm2* and *Pkm* isoforms. a, Gene report made by Swan for *Tpm2*. Relative expression of each isoform, separated by 0hr MB cells, 72hr *Pax7*^{hi} nuclei, and 72hr *Myog*^{hi} nuclei plotted alongside the isoform's name, transcript novelty, and structure. Exons 6a and 6b, known to be alternatively spliced during C2C12 differentiation, are highlighted. **b**, Gene report made by Swan for *Pkm*, separated by the same cell types. Mutually exclusive exons 9 and 10 as well as alternative TES in Pkm-205 are highlighted.

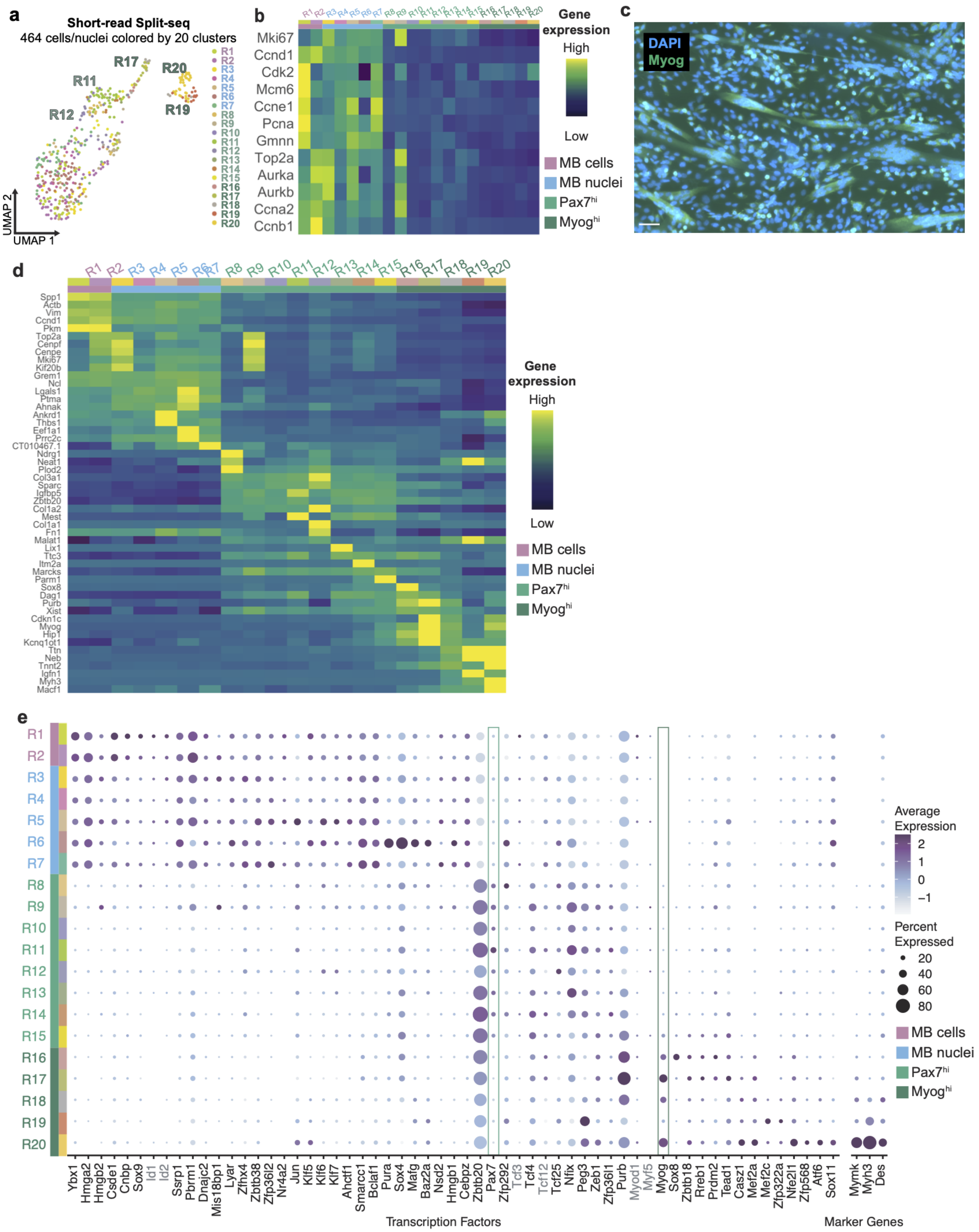

Fig. S5: Additional analysis of 38,000-cell short-read Split-seq data. a, UMAP of 464 cells with both short and long reads colored by 20 clusters derived using 36,869 short-read cells. **b**, Heatmap of cell cycle marker genes in the 20 clusters. **c**,

Visualization of *Myog* in mononucleated cells and myotubes at the 72hr differentiation timepoint. Blue = DAPI, green = *Myog*. Scale bar: 50 μm. **d**, Heatmap of marker genes in the 20 clusters (dark blue = low expression, yellow = high expression). **e**, Dot plot of transcription factors and marker genes involved in myogenesis found from differential expression testing and/or literature. Genes that did not pass the differential expression threshold yet are of interest in the system and significantly expressed in prior classic bulk data are colored grey (*Id1*, *Id2*, *Myod1*, *Myf5*, *Tcf3,* and *Tcf12*).

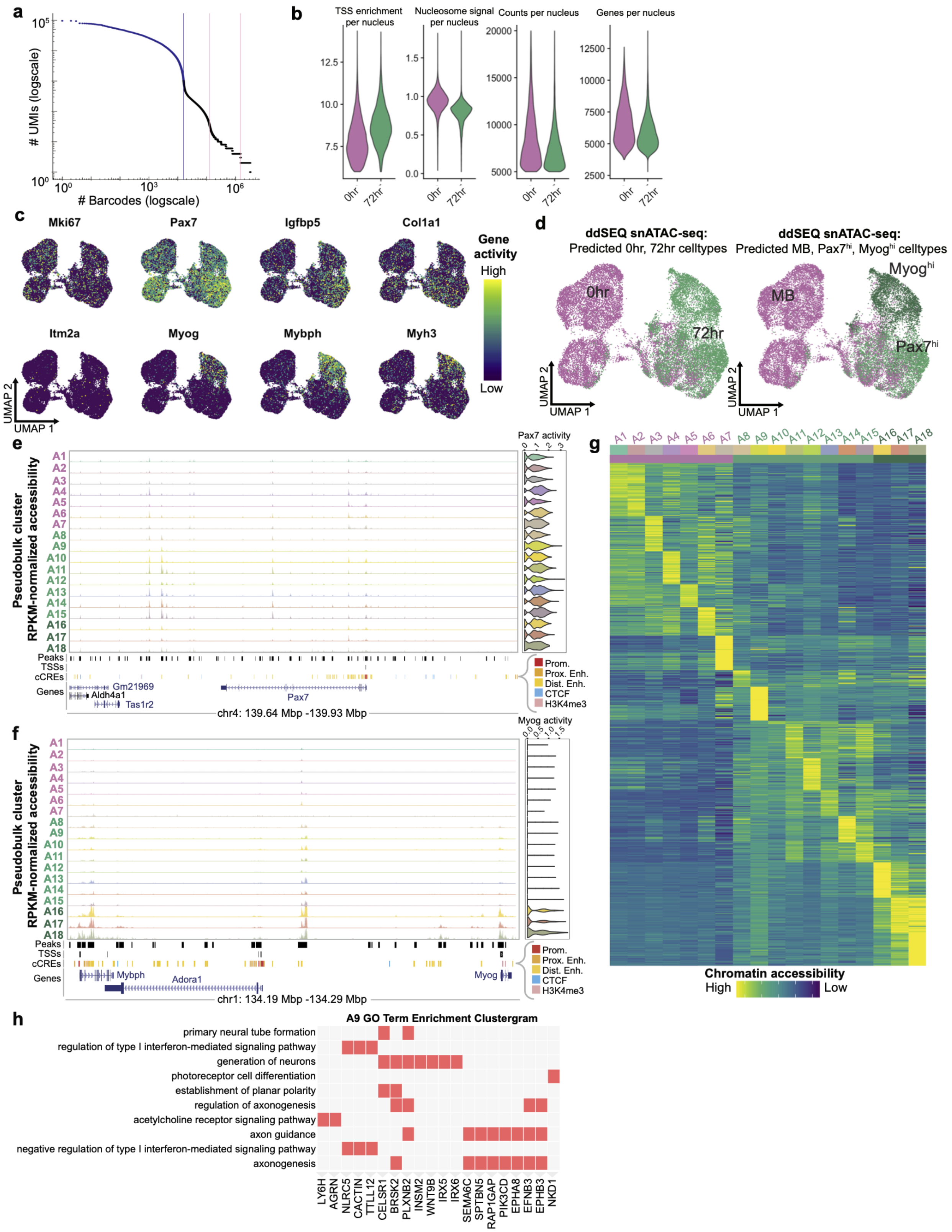

Fig. S6: Additional analysis/QC of snATAC-seq. a, UMI per barcode knee plot for an example snATAC-seq library (0hr, 6,782 nuclei). **b**, Violin plots of snATAC-seq QC

metrics after filtering > 6 TSS enrichment, < 20,000 reads, and > 5,000 reads per nucleus. **c**, Distribution of marker genes within the UMAP colored by gene activity score (dark blue = low activity, yellow = high activity). **d**, Integration of scRNA-seq and snATAC-seq data, labeled by cell type (0hr in pink and 72hr in green on left; MB in pink, *Myog*^{hi} in dark green, and *Pax7*^{hi} in light green on right). **e**, Pseudobulk peaks per cluster spanning the *Pax7* locus. TSS track indicates TSSs called from LR-Split-seq data. **f**, Pseudobulk peaks spanning the *Myog* and *Mybph* loci. **g**, Heatmap of top 50 marker regions in the 18 snATAC-seq clusters (dark blue = low accessibility, yellow = high accessibility). **h**, Cluster A9 GO term enrichment clustergram. Examples of genes associated with A9 marker peaks belonging to the GO terms in rows are indicated in red.

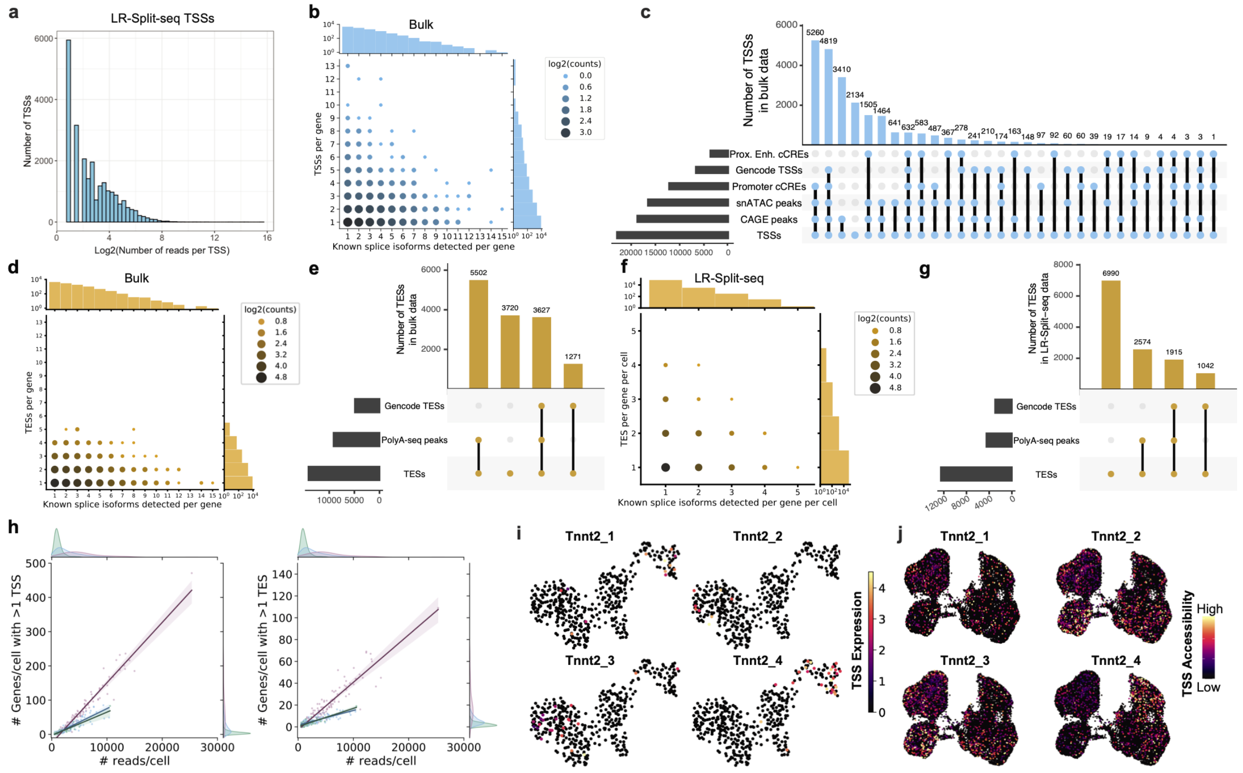

Fig. S7: Identification and validation of TSSs/TESs from long-read data. a, Histogram of number of LR-Split-seq reads supporting each TSS. **b**, Bubble plot of the number of distinct exon combinations (splice isoforms) detected per gene compared to the number of distinct TSSs detected per gene in bulk data. **c**, Validation of TSSs found in bulk long-read data using 4 external datasets (ENCODE proximal enhancer and promoter cCREs, GENCODE TSSs, and CAGE peaks) and our snATAC-seq pseudobulk peaks. **d**, Bubble plot of the number of distinct exon combinations (splice isoforms) detected per gene compared to the number of distinct TESs detected per gene found in long-read bulk data. **e**, Validation of TESs found in bulk long-reads using GENCODE TESs and polyA-seq data. **f**, Bubble plot of splice isoforms per gene per cell compared to TESs detected per gene per cell found in LR-Split-seq. **g**, Validation of

TESs found in LR-Split-seq. **h**, TSS and TES complexity (Number of genes with more than one TSS / TES per cell) vs. number of reads per cell, colored by sample type (0hr cells in pink, 0hr nuclei in blue, and 72hr nuclei in green). **i**, LR-Split-seq TSS expression for the 4 identified *Tnnt2* TSSs. **j**, snATAC accessibility for the 4 identified *Tnnt2* TSSs.

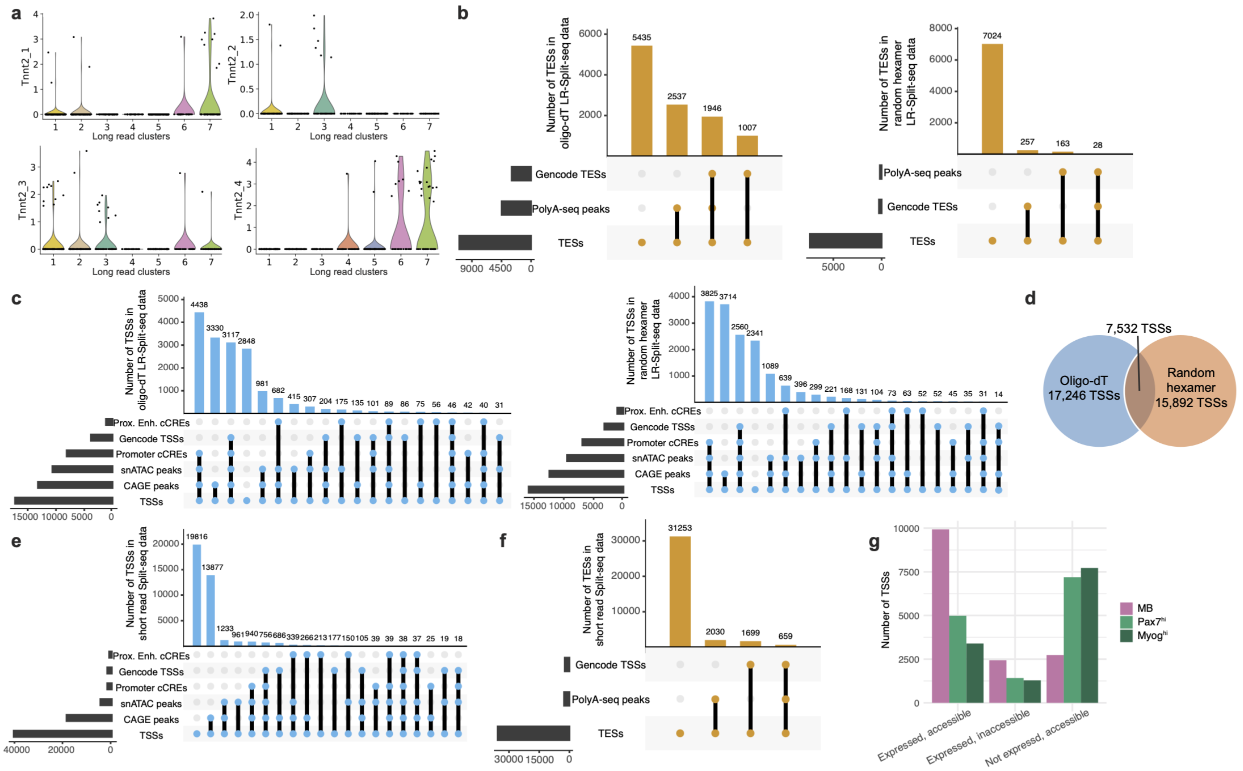

Fig. S8: Identification and validation of TSSs/TESs from oligo-dT and random hexamer long reads, and short-read data. **a**, Violin plots of TSS expression per long read cluster for the 4 identified *Tnnt2* TSSs. **b**, Validation of TESs found in LR-Split-seq data split by oligo-dT primed reads (left) and random hexamer primed reads (right). **c**, Validation of TSSs found in LR-Split-seq data split by oligo-dT primed reads (left) and random hexamer primed reads (right). **d**, Venn diagram of oligo-dT and random hexamer TSSs with 7,532 TSSs overlapping by at least 1 bp. **e**, Validation of TSSs found in short-read Split-seq data using reads fully containing the TSO (template switching oligo). **f**, Validation of TESs found in short-read Split-seq data using reads containing a 20bp poly-A sequence allowing for 50% mismatches. **g**, Comparison of thresholded expression and accessibility of TSSs split by cell type.
