# Supplementary material for: Mapping and modeling the genomic basis of differential RNA isoform expression at single-cell resolution with LR-Split-seq
Source: Genome Biol. 2021 Oct 7;22:286. doi: 10.1186/s13059-021-02505-w (PMC8495978; doi:10.1186/s13059-021-02505-w)
Supplement: Supplementary file 3 — Additional file 3:. Review history [file 13059_2021_2505_MOESM3_ESM.docx]

Review History

**First round of review**

**Reviewer 1**

**Were you able to assess all statistics in the manuscript, including the appropriateness of statistical tests used?**

Yes: Yes, I was able to assess all statistics.

**Were you able to directly test the methods?**

Yes

**Comments to author:**

The authors use myogenic differentiation as a system to validate an experimental pipeline LR-Split-seq, to generate long reads from single cells, which can identify isoform switching at the single cell or single nuclei level. The authors extensively benchmark and compare technical aspects of the protocols examined, and validate their analyses comparing to bulk long read and single nuclei accessibility. The datasets, both raw and processed data, are well curated and publicly available on GEO, as is the processing and figure generation code (GitHub). Their biological findings are well explained across a number of well annotated main text and supplementary figures.

Major comments:

1. The filtering between Fig1 J and K is mentioned in the main text but not much details on the extent of filtering, "Nevertheless, after filtering our novel transcripts with TALON, the majority of transcript models we recover are annotated in GENCODE...". The main text should list the number of isoforms that are filtered to give a sense of the efficiency. E.g. what amount of filtering brings the Known category from 4.8% of isoforms to 54.5%. The filtering also represents what proportion of the UMIs?

2. Figure S2 panel G is really useful for visualizing Tpm2 changes, this should also being included for Pkm as it is highlighted here and in the Introduction as well. Supp Tables 3 and 4 (and 12 and 13) have a column `dpi` which seems to be the change in isoform proportion, but has genes with values > 100 (all four tables), which should not be possible. Is this the max change across all the isoforms of the gene? It would be helpful to note in these tables the two transcript IDs (or TSS locations) that had the highest isoform proportion for the two conditions being compared, e.g. for Tpm2 Tpm2-203 is highest for MB and Tpm2-202 is highest for Myog^hi nuclei. This would make these results much more useful for subsequent studies.

3. Fig4 panels F and G, by looking at the data in Table S14, it was clear that the large LFC in accessibility with small changes in expression (e.g. close to the vertical dotted line) were from TSS with less than 20 reads supporting. Larger pseudocounts on the pseudo-bulk accumulated counts used to compute the fold changes or posterior log fold change estimates using the individual cell counts would likely improve this comparison.

Minor comments:

* page 6 line 7 - "to impinge differently and not entirely favorably" - phrasing awkward, suggest re-wording

* Fig1 label the slopes in panel D? Hard to read from the axis labels.

* page 8 line 36 "making the nuclear determinations less sensitive" - wasn't sure of the meaning of 'determinations' here.

* Recommend to combine Fig1 I and J with side-by-side bars for bulk and sc.

* Fig2 A and B the same color green is used to fill the bars/boxes/circles as the color used to denote 72 hr vs the other time points. A small point but is slightly distracting.

* Page 11 line NIC and NCC are not defined yet in the main text. These should be defined (also what novel in catalog and novel not in catalog refer to).

* Page 12, confusing that S1-S7 is used as S# is also referring here to supplementary figures (E.g. Fig S2 vs cluster S2).

* Fig2 panel C, why not rotate the short-read Split-seq UMAP by 180 degrees? The UMAP coordinates are arbitrary.

* Page 13, line 46: "we performed isoform switching tests with a corrected p-value cutoff from a chi-squared test of 0.05..." The Methods don't write out the details of the test, but instead cite Joglekar. Would recommend restating the details of the test as the isoform switching is key to the current manuscript. E.g. what was included for testing, what kind of p-value correction is used.

* Fig4 C, clusters A9 and A10 also have low accessibility for the promoter at TSS Tnnt2_2. Any idea what these clusters of Pax7^hi cells look like in terms of their expression?

* Supp Fig 3 and 4 could use the MB / Pax7 / Myog color legend.

* Given the processing work that went into Fig4 F-H, it would be preferable if all of the TSS with either expression or accessibility LFC outside of the dotted lines were included as a supplementary table, not solely the ones in the bottom left and top right quadrant. The other cases may be useful for subsequent investigation.

**Reviewer 2**

**Were you able to assess all statistics in the manuscript, including the appropriateness of statistical tests used?**

Yes: No concerns

**Were you able to directly test the methods?**

No

**Comments to author:**

Rebboah et al. have performed long read sequencing on Split-seq single-cell RNA-seq libraries, evaluated a number of technical considerations, applied the technology to myoblast differentiation, and integrated the data with snATAC-seq. Overall the study is important, interesting and innovative. I have several suggestions for further evaluating this useful protocol and exploring these great datasets to inform future studies.

1. The analysis of isoform information from long reads is interesting, but would benefit from a more systematic approach. Currently it comes across as very low-level and focused on specific genes, making it hard to draw generalized conclusions about biological mechanisms. For example, does each single cell express only one isoform? How much variation in isoform usage occurs across cells from the same cluster? Do isoforms vary primarily in exon usage or in 5' and 3' end selection?

2. Similarly, the evaluation of TSS usage using both RNA and ATAC information is fascinating, but it should be more systematic and genomewide, rather than anecdotal. This dataset provides an incredible opportunity to explore fundamental questions of regulation. For example, how accurately does the accessibility profile of a TSS within a cell or cell type predict the transcription from the same TSS? How much does the TSS accessibility predict the exon usage downstream of the TSS? Is there any correlation between accessibility of peaks near a gene and its isoform usage?

3. The availability of long-read data and short-read data from the same cell/nucleus provides an unprecedented opportunity to investigate the accuracy of the spliced/unspliced counts used for RNA velocity analysis. How similar/different are the pre-mRNA vs. mature mRNA counts as calculated from short read vs. long read data?

4. Can the random hexamer priming reads from LR-split-seq detect non-polyA lncRNA transcripts that would be missed with conventional 3' end scRNA protocols? If so, this is important to report and explore.

**Reviewer 2**

**Were you able to assess all statistics in the manuscript, including the appropriateness of statistical tests used?**

No

**Were you able to directly test the methods?**

No

**Comments to author:**

In the manuscript, Rebboah et al. introduced a new method (namely "LR-Split-seq") to generate PacBio long-read sequencing library for "split-and-pool" strategy based single-cell RNA-seq method Split-seq. With the combined sequencing approach using PacBio long-read sequencing and Illumina short-read platform, the authors identified cell-type-specific RNA isoform expression landscape with a specialized focus on alternative splicing, and alternative TSS usage in the mouse C2C12 system. However, as a method paper, the manuscript lacks the significance in term of method development. The main approach of this method is to separate the libraries for long-read and short-read sequencing.

Based on the name of the method, I was expecting that the authors will focus on improving LR detection and more importantly, showing the advantage of LR sequencing. However, the comparison between SR sequencing and LR sequencing was lacking in many analyses. For example, in TES analysis, the authors sequenced the long-read library of 464 cells, and short-read library of ~37k cells. Even with short-read sequencing, I will expect to distinguish several of the alternative splicing transcripts. It is worth noting that the short-read Split-seq library sequences the 3' end, therefore it is perfect for TES analysis. The authors could evaluate the performance of TES analysis by short-read sequencing. I suspect that the large number of cells may compensate for the inefficient characterization of splicing isoforms. It is desired to address whether the short-read based TES analysis yield similar results to the bulk, or LR-Split-seq. Here is another case, in the single-nucleus read length (Fig. 1f), 1/3 of the reads are shorter than 600 bp, meaning that they are qualified for Illumina short-read sequencing. If the LR-Split-seq cannot be adapted for large-scale sequencing, these 1/3 of short reads could be completely sequenced on the Illumina platform, and provide the TSS information as well. Could the authors evaluate the performance of TSS prediction using the current short-read data?

I also noticed that authors only sequenced a limited number of long-read libraries. I am not sure why only limited number of cells were sequenced with LR. If it is technological or economical challenging to improve the throughput, then it is important to show that even with limited number of cells, LR-split-seq can still capture new information that the large-scale short-read sequencing yield cannot.

Below are sone minor points that demands more supportive evidence or additional analysis.

1. The authors observed that single-nucleus LR-Split-seq yields shorter reads compared to single-cell LR-Split-seq (Fig. 1f). Next, the authors attribute the read length differences between single-nucleus and single-cell LR-Split-seq to the different proportions of mature RNA. This explanation does not make sense to me. If the authors' speculation is true, I would expect that longer cDNA products can be produced from nascent RNA than the mature RNA, as a result, we would expect the opposite trend of read length. I think the reason for this observation is that nucleus is more compact than cytoplasm, therefore, the same fixation protocol could generate stronger crosslinking in the nuclei, which then lead to shorter amplicon size.

2. On page 13, line 51, the authors stated that "vast majority of novel transcript models were only reproducible between bulk replicates". Some NIC and NNC transcripts are shared by at one LR-Split-seq sample and at least one bulk, which indicate that these isoforms could be true; but what about the large number of novel transcripts that are only detected in one LR-Split-seq sample? An evaluation of false positive rate is desired. Showing that LR-Split-seq alone can effectively and robustly identify novel transcripts can significantly increase the value of this method.

3. The Venn diagrams in Fig. S1h and S1i suggest that LR-Split-seq only detected very limited numbers of novel transcripts. Is this due to inadequate total read depth, or potentially large heterogeneity between single cells?

4. On page 25, line 22 "We validated genome-wide TESs using GENCODE vM21 TESs and polyA-seq peaks from C2C12 cells at days 0 and 4 of differentiation, which overlapped the majority of TESs found in bulk data but not in the LR-Split-seq data." Does this indicate there are a large number of false positives in splicing-isoform-specific TESs identified by LR-Split-seq?

**Reviewer 4**

**Were you able to assess all statistics in the manuscript, including the appropriateness of statistical tests used?**

Yes: There weren't many statistical results but I looked at the code to reproduce the data plots.

**Were you able to directly test the methods?**

No

**Comments to author:**

Current approaches to long-read scRNA-Seq require microfluidics approaches that do not allow easy or affordable scaling to very large numbers of cells. By adapting Split-Seq for use with long-read sequencing the authors have provides a very useful experimental approach for scaling up long-read single-cell RNA-Seq. They benchmark against long-read bulk data and against short read scRNA-Seq data, and also integrate their results with sn-ATAC-Seq to help interpret the alternative TSS results in their exemplar application.

The technical validation of the method looks very thorough. The comparison with bulk long-read sequencing shows reasonable concordance at the gene level (79%) and about half (53%) of bulk-identified isoforms are found. This lower level of isoform detection is attributed to use of only full-length reads for isoform detection versus gene detection which uses all reads. That makes sense and suggests there may be computational approaches to increase isoform detection levels for this technology (but perhaps at the cost of less certainty in isoform identification) through deconvolution similar to short-read technologies. For example, perhaps there are incomplete reads that still provide good evidence of TSS switching.

The experimental results are interesting, showing clear examples of changes in TSS usage within cell clusters across conditions that would be impossible with standard scRNA-Seq, while pinning changes down to cell clusters would be impossible with bulk, so the single-cell resolution is clearly an advantage in terms of interpretability.

The fact that the technology works with single nucleus data is very encouraging since for many samples it will be impossible to disassociate cells.

The paper is well written and clear. I would particularly like to comment on the high quality of reproducibility in the data analysis. Every figure is accompanied by code to reproduce all the figure panels and the code is really nicely formatted to be broken up into understandable chunks. This is excellent and makes it much easier to check the computational methods.

Minor comment:

Caption to Figure 1 in the first sentence should also mention short-read Split-Seq I think, it only mentions LR-Split-Seq.

**Authors Response**

**Point-by-point responses to the reviewers’ comments:**

*We thank all of the reviewers for their comments and we have included a point-by point response below starting with the heading “Response:”. In addition, we have revised the abstract to fit within 100 words, as well as addressed the Editor’s request to state the open source license in the text with accession links in Github and Zenodo.*

Reviewer #1: The authors use myogenic differentiation as a system to validate an experimental pipeline LR-Split-seq, to generate long reads from single cells, which can identify isoform switching at the single cell or single nuclei level. The authors extensively benchmark and compare technical aspects of the protocols examined, and validate their analyses comparing to bulk long read and single nuclei accessibility. The datasets, both raw and processed data, are well curated and publicly available on GEO, as is the processing and figure generation code (GitHub). Their biological findings are well explained across a number of well annotated main text and supplementary figures.

*Response: We thank the reviewer for his overall comments and answer his specific comments below.*

Major comments:

1. The filtering between Fig1 J and K is mentioned in the main text but not much details on the extent of filtering, "Nevertheless, after filtering our novel transcripts with TALON, the majority of transcript models we recover are annotated in GENCODE...". The main text should list the number of isoforms that are filtered to give a sense of the efficiency. E.g. what amount of filtering brings the Known category from 4.8% of isoforms to 54.5%. The filtering also represents what proportion of the UMIs?

*Response: The reviewer is correct that this would be very informative. We retain 40,982 isoforms, which correspond to 34.8% of the reads and 34.5% of the UMIs. We have added this to page 9 of the manuscript.*

2. Figure S2 panel G is really useful for visualizing Tpm2 changes, this should also being included for Pkm as it is highlighted here and in the Introduction as well. Supp Tables 3 and 4 (and 12 and 13) have a column `dpi` which seems to be the change in isoform proportion, but has genes with values > 100 (all four tables), which should not be possible. Is this the max change across all the isoforms of the gene? It would be helpful to note in these tables the two transcript IDs (or TSS locations) that had the highest isoform proportion for the two conditions being compared, e.g. for Tpm2 Tpm2-203 is highest for MB and Tpm2-202 is highest for Myog^hi nuclei. This would make these results much more useful for subsequent studies.

*Response: We thank their reviewer for the suggestion and appreciation for existing visualizations. We have added a Swan plot for Pkm as well in Figure S3 panel A as well as expanded the background about which exons are seen to be implicated in the switch, which are also labelled in the new Swan plot.*

*For each testable gene, Δπ, or the change in percent isoform usage, was computed as the sum of the top two differences in percent isoform usage across the conditions. For this reason, it is possible for two percent isoform changes (for two distinct isoforms of the same gene) to sum up to over 100. We have added this explanation to the methods section.*

*We have also added 4 columns to the tables output from the isoform / TSS switching tests. Originally, we tried to do exactly as the reviewer suggested and include the top isoform from either condition. However, this yielded many entries where the top isoform is actually the same in each of the conditions, which implies that the isoform switch is actually driven by the change in what percentage the top isoform is expressed in each condition and by the same changes in the second most abundant isoform, by the definition of the test. Therefore, we have included columns for the top two abundant isoforms across both conditions (which are the isoforms that are used to calculate the gene’s Δπ value), as well as the isoforms’ individual Δπ values, to demonstrate the exact basis on which the isoform switch was called.*

3. Fig4 panels F and G, by looking at the data in Table S14, it was clear that the large LFC in accessibility with small changes in expression (e.g. close to the vertical dotted line) were from TSS with less than 20 reads supporting. Larger pseudocounts on the pseudo-bulk accumulated counts used to compute the fold changes or posterior log fold change estimates using the individual cell counts would likely improve this comparison.

*Response: At the reviewer’s suggestion, we tried adding several pseudocounts to our plots for Fig 4 F/G and did not find any substantial change to the comparisons. We therefore elected to leave them as is.*

Minor comments:

* page 6 line 7 - "to impinge differently and not entirely favorably" - phrasing awkward, suggest re-wording

*Response: We changed this sentence, which is now on page 5, to read “However, this benefit in the short-read format is expected to have a different and unfavorable effect in long-read data.”*

* Fig1 label the slopes in panel D? Hard to read from the axis labels.

*Response: We have added regression lines to figures 1D and E as well as reported the slopes of the regression lines in the legend.*

* page 8 line 36 "making the nuclear determinations less sensitive" - wasn't sure of the meaning of 'determinations' here.

*Response: we have taken the word determination out. This now reads as “making the nuclei less sensitive than whole cells”.*

* Recommend to combine Fig1 I and J with side-by-side bars for bulk and sc.

*Response: While we appreciate the reviewer’s comment, we feel that separate panels 1i and 1j, which are next to each other, allow the readers to look at the two separate Y-axes of reads and read immediately how many long-reads are in each column of 1j. This would be hard to do if the columns of 1j are on the same scale as 1i and would be more confusing with a second Y-axis to the right in a combined panel.*

* Fig2 A and B the same color green is used to fill the bars/boxes/circles as the color used to denote 72 hr vs the other time points. A small point but is slightly distracting.

*Response: We used this color to represent the “known” category for transcript and gene novelty, which is a convention we have used across all of our long-read manuscripts. We would prefer to keep this scheme consistent across our manuscripts.*

* Page 11 line NIC and NCC are not defined yet in the main text. These should be defined (also what novel in catalog and novel not in catalog refer to).

*Response: The reviewer is correct that these terms needed further explanation for those unfamiliar to the terminology. We added a section on page 6 when we first mention the analysis performed using TALON to briefly explain these categories.*

* Page 12, confusing that S1-S7 is used as S# is also referring here to supplementary figures (E.g. Fig S2 vs cluster S2).

*Response: We thank the reviewer for his comment and have changed throughout the text the short read cluster names from S# to SR# and the corresponding long read cluster names from L# to LR# such that they match.*

* Fig2 panel C, why not rotate the short-read Split-seq UMAP by 180 degrees? The UMAP coordinates are arbitrary.

*Response: We thank the reviewer for his comment and have rotate Panel C by 180 degrees, which indeed makes it easier to compare to Figure 2D and 2E.*

* Page 13, line 46: "we performed isoform switching tests with a corrected p-value cutoff from a chi-squared test of 0.05..." The Methods don't write out the details of the test, but instead cite Joglekar. Would recommend restating the details of the test as the isoform switching is key to the current manuscript. E.g. what was included for testing, what kind of p-value correction is used.

*Response: We thank the reviewer for pointing out this point of confusion for future readers. As stated in the above response to the reviewer’s major comment, we have added methods text about the isoform switching analysis.*

* Fig4 C, clusters A9 and A10 also have low accessibility for the promoter at TSS Tnnt2_2. Any idea what these clusters of Pax7^hi cells look like in terms of their expression?

*Response: Based on the reviewer’s advice, we looked into GO terms associated with the genes near the chromatin peaks in these clusters. Interestingly, A9 revealed a number of possible NMJ-related GO terms. A10 did not show any interesting GO terms and it is therefore difficult to assess what that cluster is doing. We have added the A9 GO analysis to the results section along with a GO term enrichment chart as well as clustergram to the supplement to highlight what this particular cluster might be involved in (Fig S6H).*

* Supp Fig 3 and 4 could use the MB / Pax7 / Myog color legend.

*Response: We added the color legend for these figures.*

* Given the processing work that went into Fig4 F-H, it would be preferable if all of the TSS with either expression or accessibility LFC outside of the dotted lines were included as a supplementary table, not solely the ones in the bottom left and top right quadrant. The other cases may be useful for subsequent investigation.

*Response: We have now added this to our supplementary table.*

Reviewer #2: Rebboah et al. have performed long read sequencing on Split-seq single-cell RNA-seq libraries, evaluated a number of technical considerations, applied the technology to myoblast differentiation, and integrated the data with snATAC-seq. Overall the study is important, interesting and innovative. I have several suggestions for further evaluating this useful protocol and exploring these great datasets to inform future studies.

*Response: We thank the reviewer for their overall comments and have revised our manuscript to address their suggestions, as elaborated below.*

1. The analysis of isoform information from long reads is interesting, but would benefit from a more systematic approach. Currently it comes across as very low-level and focused on specific genes, making it hard to draw generalized conclusions about biological mechanisms. For example, does each single cell express only one isoform? How much variation in isoform usage occurs across cells from the same cluster? Do isoforms vary primarily in exon usage or in 5' and 3' end selection?

*Response: We have taken the reviewer’s suggestion to heart and examined the isoform complexity of each cell by counting the number of genes that express multiple isoforms from the same single cell or nucleus. Only one isoform was typically detected per gene in each cell. The number of genes expressing more than one isoform is a linear function of read depth per cell, suggesting that deeper sequencing will increase isoform complexity per gene (Fig S3D). Furthermore, we noticed a clear difference in the relationship between isoform complexity and read depth when comparing the single cells with single nuclei where the nuclei of increasing read depth do not display a similar large increase in isoform complexity as do the cells (Fig S3D). One explanation for this is that LR-Split-seq of a nucleus captures a snapshot of its immediate state of splicing, whereas LR-Split-seq in cells captures the sum of different isoforms produced and exported to the cytoplam over a longer period of time. If correct, the implication is that splicing within the nucleus is transiently biased for one pattern, and conceivably for one allele, the identity of which changes dynamically. We have added these findings on page 14 of the manuscript.*

*We performed the same complexity analysis on the identified TSSs and TESs per gene per cell that we did on the isoform level. We found nearly identical results where the cells and nuclei with more reads have a higher number of genes that express more than one TSS or TES and that the cells exhibit more TSS or TES complexity overall (Fig. S7H). We have added these findings on page 23 of the manuscript.*

2. Similarly, the evaluation of TSS usage using both RNA and ATAC information is fascinating, but it should be more systematic and genomewide, rather than anecdotal. This dataset provides an incredible opportunity to explore fundamental questions of regulation. For example, how accurately does the accessibility profile of a TSS within a cell or cell type predict the transcription from the same TSS? How much does the TSS accessibility predict the exon usage downstream of the TSS? Is there any correlation between accessibility of peaks near a gene and its isoform usage?

*Response: We calculated snATAC TSS chromatin accessibility across our refined set of TSSs to determine how TSS accessibility relates to TSS expression. We found that binary chromatin accessibility at a TSS was a particularly strong indicator of whether or not a TSS was expressed in MBs (75% of TSSs) and that the level of accessibility at each TSS correlated well with the expression level of each TSS (Pearson r = 0.44, Spearman rho = 0.58). In the Pax7hi and Myoghi populations, accessibility at each TSS did not correlate as strongly (Pearson r = 0.58, Spearman rho = 0.20; Pearson r = 0.53, Spearman rho = 0.16 respectively). We then determined how many genes that we called more than one TSS for displayed the highest accessibility level and expression level at the same TSS. Again, for the MBs, the most highly-accessible TSS for a gene was most often also the most highly-expressed TSS for the gene (77.8% of genes with >1 TSS). This concordance was less strong in the Pax7hi and Myoghi groups (54.6% and 52.7% respectively. We have added this to page 25 of the manuscript.*

3. The availability of long-read data and short-read data from the same cell/nucleus provides an unprecedented opportunity to investigate the accuracy of the spliced/unspliced counts used for RNA velocity analysis. How similar/different are the pre-mRNA vs. mature mRNA counts as calculated from short read vs. long read data?

*Response: We thank the reviewer for his suggestion. We ran Velocyto on the short-read and long-read datasets for our 464 cells. We found remarkably similar patterns of RNA velocity across the short and long read. Furthermore, the ratio of spliced / unspliced reads was higher in short vs. long (though still very similar). We have added this to page 13 of the manuscript as well as Fig S3B and S3C.*

4. Can the random hexamer priming reads from LR-split-seq detect non-polyA lncRNA transcripts that would be missed with conventional 3' end scRNA protocols? If so, this is important to report and explore.

*Response: We agree with the reviewer that this would have been an exciting result. we looked for the detection and differential expression of histone genes (https://doi.org/10.1186/s13072-016-0109-x), which are known to be non-polyadenylated, as well as a set of experimentally-confirmed genes that are non-polyadenylated (from https://doi.org/10.1186/gb-2011-12-2-r16) in our random hexamer primed reads and oligo dT primed reads from our LR-Split-seq data. Overall, while there were more histone and non-polyadenylated genes detected in the random hexamer reads versus the oligo dT reads, none of these genes were found to be significantly upregulated in the random hexamer reads versus the oligo dT reads. We cannot therefore recommend random priming as a robust capture method for detecting non-polyadenylated genes.*

Reviewer #3: In the manuscript, Rebboah et al. introduced a new method (namely "LR-Split-seq") to generate PacBio long-read sequencing library for "split-and-pool" strategy based single-cell RNA-seq method Split-seq. With the combined sequencing approach using PacBio long-read sequencing and Illumina short-read platform, the authors identified cell-type-specific RNA isoform expression landscape with a specialized focus on alternative splicing, and alternative TSS usage in the mouse C2C12 system. However, as a method paper, the manuscript lacks the significance in term of method development. The main approach of this method is to separate the libraries for long-read and short-read sequencing.

*Response: We would like to point out that we have substantial computational methods development in this manuscript, as the demultiplexing of Split-seq long-reads given the higher indel rate of long-reads (compared to Illumina short-reads) and the mixture of priming strategies is more challenging than the reviewer assumes.*

Based on the name of the method, I was expecting that the authors will focus on improving LR detection and more importantly, showing the advantage of LR sequencing. However, the comparison between SR sequencing and LR sequencing was lacking in many analyses. For example, in TES analysis, the authors sequenced the long-read library of 464 cells, and short-read library of ~37k cells. Even with short-read sequencing, I will expect to distinguish several of the alternative splicing transcripts. It is worth noting that the short-read Split-seq library sequences the 3' end, therefore it is perfect for TES analysis.

*Response: the reviewer is partially correct that the Illumina reads potentially could be mined for TES, with the caveat that this is only true for the oligo-dT primers and not the random hexamer priming. Unfortunately, the standard Split-seq pipelines merge the random-priming reads for each cell into the oligo-dT barcodes (i.e. they report all reads with the oligo-dT barcodes), and it may be that random priming accounts for the poor performance at the TES for Illumina. In particular, most of the Illumina reads do not have evidence of polyA and therefore are less informative than the reviewer assumes.*

The authors could evaluate the performance of TES analysis by short-read sequencing. I suspect that the large number of cells may compensate for the inefficient characterization of splicing isoforms. It is desired to address whether the short-read based TES analysis yield similar results to the bulk, or LR-Split-seq. Here is another case, in the single-nucleus read length (Fig. 1f), 1/3 of the reads are shorter than 600 bp, meaning that they are qualified for Illumina short-read sequencing. If the LR-Split-seq cannot be adapted for large-scale sequencing, these 1/3 of short reads could be completely sequenced on the Illumina platform, and provide the TSS information as well. Could the authors evaluate the performance of TSS prediction using the current short-read data?

*Response: The reviewer speculates that we could sequence most of the transcripts less than 600 bp on an Illumina platform, which is only partially true. All of the high-throughput platforms for Illumina (NovaSeq, NextSeq) are limited to 2x150bp formats, with a severe degradation in read quality past 125bp on each end. While the miSeq platform does support 2x300bp format, it is limited to ~20 million reads (https://www.illumina.com/systems/sequencing-platforms.html). In any case, 86bp of one of the read ends would be used for the barcode. We do not think that a 20 million Illumina read experiment would be acceptable to the community and as pointed out by the reviewer, it would only work for a subset of the transcripts (which turn out to be mostly intronic reads in the single-nuclei data, anyhow).*

*Based on the reviewer suggestions, we compared how well our long-reads are supported by external validation in comparison to those we called with from our short-read Split-seq data for the same cells using analogous approaches. For the TSSs, we subset the input short reads on those that contained the template-switching oligo (TSO) sequence which in theory should represent the 5’ end of the transcript. Using this subset of reads, we ran the same peak calling script to call TSSs and required that each one was supported by at least 20 reads. Strikingly, we found that compared to the 81.5% of TSSs that had external validation from LR-Split-seq, only 50.2% of TSSs called using the short reads had external validation. For the short-read Split-seq TESs, to limit the analysis only to reads that contain the 3’ end of the transcript, we subset the input short reads on those that contained a run of 20bp where at least half of the bases were A and required each TES to be supported by at least 20 short reads. The short-read Split-seq TESs validate externally at a much lower rate (12.3%) than the LR-Split-seq TESs (44.2%). We have added these to page 25 of the manuscript as well as in supplemental Figure S8.*

I also noticed that authors only sequenced a limited number of long-read libraries. I am not sure why only limited number of cells were sequenced with LR. If it is technological or economical challenging to improve the throughput, then it is important to show that even with limited number of cells, LR-split-seq can still capture new information that the large-scale short-read sequencing yield cannot.

*Response: The reviewer is correct that we can of course sequence as many cells as we want using any platform, but we think that this restricts long-read sequencing to a small number of labs with large budgets. The key idea is to have a relatively high number of reads per cell/nuclei in order to detect a decent number of genes and transcript isoforms. While most modern high-throughput single-cell capture platforms can capture 10,000+ cells per experiment, one of the great strengths of Split-seq is the ability to build multiple sub-libraries from the same labeled pool with different numbers of cells. A conservative estimate of 4 million CCS reads per SMRT cell would mean 400 reads / cell on average for a regular 10x experiment with 10,000 cells and 4,000 reads /cell for a Split-seq sublibrary while still being able to sequence 37,000 cells on Illumina from the same labeled pool. We could have selected more cells per sublibrary, but a priori there was no reason to expect many cell subtypes from a single, well-studied cell line being differentiated.*

Below are sone minor points that demands more supportive evidence or additional analysis.

1. The authors observed that single-nucleus LR-Split-seq yields shorter reads compared to single-cell LR-Split-seq (Fig. 1f). Next, the authors attribute the read length differences between single-nucleus and single-cell LR-Split-seq to the different proportions of mature RNA. This explanation does not make sense to me. If the authors' speculation is true, I would expect that longer cDNA products can be produced from nascent RNA than the mature RNA, as a result, we would expect the opposite trend of read length. I think the reason for this observation is that nucleus is more compact than cytoplasm, therefore, the same fixation protocol could generate stronger crosslinking in the nuclei, which then lead to shorter amplicon size.

*Response: We respectfully disagree with the reviewer. Spliced mRNA is a smaller proportion of the overall amount of RNA molecules found at any given moment in the nucleus, since for every spliced mRNA with N exons that we detect, there are N-1 additional lariats that will also persist for a few minutes within the nucleus. Many of these lariats in mammals will be longer than mature mRNAs. The relative dearth of isoforms with intron retention events and the preponderance of genomic-only reads that do not overlap exons strongly suggests that there are plenty of intron lariats that are present in the nucleus at any given time to be primed (especially by random priming, but there will be plenty of poly(A) runs in those introns).*

2. On page 13, line 51, the authors stated that "vast majority of novel transcript models were only reproducible between bulk replicates". Some NIC and NNC transcripts are shared by at one LR-Split-seq sample and at least one bulk, which indicate that these isoforms could be true; but what about the large number of novel transcripts that are only detected in one LR-Split-seq sample? An evaluation of false positive rate is desired. Showing that LR-Split-seq alone can effectively and robustly identify novel transcripts can significantly increase the value of this method.

*Response: We have a high threshold of reproducibility for novel isoforms (as discussed in the next response). Per the reviewer’s suggestion, we evaluated whether our novel isoforms validate in the bulk samples to calculate a true positive rate by category. Focusing on the two major classes of full-length novelty, we find a minimum TPRs of 0.77 for NIC and 0.59 for NNC. We note that the calculated TPR for NIC based on the bulk is higher than for known (0.71) and that it is certainly possible that an additional subset of the NIC/NNC isoforms discovered in LR-split-seq were missed in the bulk because they are lowly expressed. We have now added this to page 12 of the manuscript.*

3. The Venn diagrams in Fig. S1h and S1i suggest that LR-Split-seq only detected very limited numbers of novel transcripts. Is this due to inadequate total read depth, or potentially large heterogeneity between single cells?

*Response: The TALON filter works based on transcript reproducibility between datasets. On the single cell level, this translates into reproducibility between barcoded single cells. For LR-Split-seq, we used a very strict filter, which required that each novel isoform was reproducible in at least 4 different barcoded cells.; This approach emphasizes specificity over sensitivity (especially as the underlying assay, long-read RNA sequencing, has sensitivity issues of its own). In future experiments, the strictness of the filter and the depth of sequencing of LR-Split-seq libraries can be modified. But for this experiment, the strictness of the filter gives us confidence that the novel transcripts we are allowing through the filter are real transcripts. Last but not least, C2C12 is a very extensively-studied cell line and we do not expect to see a high number of novel isoforms.*

4. On page 25, line 22 "We validated genome-wide TESs using GENCODE vM21 TESs and polyA-seq peaks from C2C12 cells at days 0 and 4 of differentiation, which overlapped the majority of TESs found in bulk data but not in the LR-Split-seq data." Does this indicate there are a large number of false positives in splicing-isoform-specific TESs identified by LR-Split-seq?

*Response: We believe that the apparent lack of external validation for the LR-Split-seq TESs is largely driven by the random priming method. When we call TESs instead using the same set of reads but split by priming strategy, 50.3% of the oligo-dT TESs validate by at least one form of external support compared to 6% of the random hexamer TESs (Fig. S8B). We have added this to page 25 of the manuscript.*

Reviewer #4: Current approaches to long-read scRNA-Seq require microfluidics approaches that do not allow easy or affordable scaling to very large numbers of cells. By adapting Split-Seq for use with long-read sequencing the authors have provides a very useful experimental approach for scaling up long-read single-cell RNA-Seq. They benchmark against long-read bulk data and against short read scRNA-Seq data, and also integrate their results with sn-ATAC-Seq to help interpret the alternative TSS results in their exemplar application.

The technical validation of the method looks very thorough. The comparison with bulk long-read sequencing shows reasonable concordance at the gene level (79%) and about half (53%) of bulk-identified isoforms are found. This lower level of isoform detection is attributed to use of only full-length reads for isoform detection versus gene detection which uses all reads. That makes sense and suggests there may be computational approaches to increase isoform detection levels for this technology (but perhaps at the cost of less certainty in isoform identification) through deconvolution similar to short-read technologies. For example, perhaps there are incomplete reads that still provide good evidence of TSS switching.

The experimental results are interesting, showing clear examples of changes in TSS usage within cell clusters across conditions that would be impossible with standard scRNA-Seq, while pinning changes down to cell clusters would be impossible with bulk, so the single-cell resolution is clearly an advantage in terms of interpretability.

The fact that the technology works with single nucleus data is very encouraging since for many samples it will be impossible to disassociate cells.

The paper is well written and clear. I would particularly like to comment on the high quality of reproducibility in the data analysis. Every figure is accompanied by code to reproduce all the figure panels and the code is really nicely formatted to be broken up into understandable chunks. This is excellent and makes it much easier to check the computational methods.

*Response: We thank the reviewer for their comments and think that they would find the revised manuscript to be even more interesting.*

Minor comment:

Caption to Figure 1 in the first sentence should also mention short-read Split-Seq I think, it only mentions LR-Split-Seq.

*Response: we have now added short-read split-seq to the legend.*

**Second round of review**

**Reviewer 1**

The authors' revision has addressed all my previous comments.

**Reviewer 2**

I commend the authors on their thoughtful and thorough responses to my comments. I have no further concerns. Congratulations on an interesting study.
